# Intestine‐Specific Overexpression of Carboxylesterase 2c Protects Mice From Diet‐Induced Liver Steatosis and Obesity

**DOI:** 10.1002/hep4.1292

**Published:** 2018-12-17

**Authors:** Lisa Katharina Maresch, Pia Benedikt, Ursula Feiler, Sandra Eder, Kathrin A. Zierler, Ulrike Taschler, Stephanie Kolleritsch, Thomas O. Eichmann, Gabriele Schoiswohl, Christina Leopold, Beatrix I. Wieser, Caroline Lackner, Thomas Rülicke, Jan van Klinken, Dagmar Kratky, Tarek Moustafa, Gerald Hoefler, Guenter Haemmerle

**Affiliations:** ^1^ Institute of Molecular Biosciences University of Graz Graz Austria; ^2^ Gottfried Schatz Research Center, Molecular Biology and Biochemistry Medical University of Graz Graz Austria; ^3^ Diagnostic & Research Center for Molecular BioMedicine Institute of Pathology Medical University of Graz Graz Austria; ^4^ Institute of Laboratory Animal Science University of Veterinary Medicine Wien Austria; ^5^ Department of Human Genetics Leiden University Medical Centre Leiden the Netherlands; ^6^ Division of Gastroenterology and Hepatology Medical University Graz Graz Austria

## Abstract

Murine hepatic carboxylesterase 2c (*Ces2c*) and the presumed human ortholog carboxylesterase 2 (*CES2*) have been implicated in the development of nonalcoholic fatty liver disease (NAFLD) in mice and obese humans. These studies demonstrated that *Ces2c* hydrolyzes triglycerides (TGs) in hepatocytes. Interestingly, *Ces2c*/*CES2* is most abundantly expressed in the intestine, indicating a role of *Ces2c*/*CES2* in intestinal TG metabolism. Here we show that *Ces2c* is an important enzyme in intestinal lipid metabolism in mice. Intestine‐specific *Ces2c* overexpression (Ces2c^int^) provoked increased fatty acid oxidation (FAO) in the small intestine accompanied by enhanced chylomicron clearance from the circulation. As a consequence, high‐fat diet–fed Ces2c^int^ mice were resistant to excessive diet‐induced weight gain and adipose tissue expansion. Notably, intestinal *Ces2c* overexpression increased hepatic insulin sensitivity and protected mice from NAFLD development. Although lipid absorption was not affected in Ces2c^int ^mice, fecal energy content was significantly increased. Mechanistically, we demonstrate that *Ces2c* is a potent neutral lipase, which efficiently hydrolyzes TGs and diglycerides (DGs) in the small intestine, thereby generating fatty acids (FAs) for FAO and monoglycerides (MGs) and DGs for potential re‐esterification. Consequently, the increased availability of MGs and DGs for re‐esterification and primordial apolipoprotein B_48_ particle lipidation may increase chylomicron size, ultimately mediating more efficient chylomicron clearance from the circulation. *Conclusion:* This study suggests a critical role for Ces2c in intestinal lipid metabolism and highlights the importance of intestinal lipolysis to protect mice from the development of hepatic insulin resistance, NAFLD, and excessive diet‐induced weight gain during metabolic stress.

**Abbreviations**
AEE: activity‐based energy expenditure
Acox: acyl‐CoA oxidase
ANCOVA: analysis of covariance
apoB_48_: apolipoprotein B_48_
ASMs: acid‐soluble metabolites
CD36: cluster of differentiation 36
Ces1g: carboxylesterase 1g (murine)
CES2: carboxylesterase 2 (human)
Ces2c: carboxylesterase 2c (murine)
Ces2c^int^: intestine‐specific Ces2c overexpressing (mice)
CGI‐58: comparative gene identification‐58
CoA: coenzyme A
Cpt1a: carnitine palmitoyl‐transferase *1 alpha*
Cpt1b: carnitine palmitoyl‐transferase 1 beta
DG: diglyceride
Dgat1: diacylglycerol acyltransferase 1
DGH: diglyceride hydrolase
EE: energy expenditure
ER: endoplasmic reticulum
 FA: fatty acid
FAO: fatty acid oxidation
FFA: free fatty acid
FPLC: fast protein liquid chromatography
HFD: high‐fat diet
LD: lipid droplet
Lpl: lipoprotein lipase
MG: monoglyceride
mRNA: messenger RNA
Mttp: microsomal triglyceride transfer protein
NAFLD: nonalcoholic fatty liver disease
OA: oleic acid
OCR: oxygen consumption rate
OFTT: oral fat tolerance test
PA: palmitic acid
Ppara: peroxisome proliferator‐activated receptor alpha
RER: respiratory exchange rate
RMR: resting metabolic rate
TEE: total energy expenditure
TG: triglyceride
TGH: triglyceride hydrolase
VLDL: very low density lipoprotein
WT: wild type

The epidemic prevalence of obesity constitutes one of the greatest public health challenges of the 21st century. Obese individuals are at high risk of developing cardiovascular diseases, type 2 diabetes, and nonalcoholic fatty liver disease (NAFLD), which is the most common chronic liver disease worldwide. Dyslipidemia is a prominent characteristic of obese individuals and patients with NAFLD. The onset of dyslipidemia is tightly linked to an aberrant production and/or turnover of triglyceride (TG)‐rich lipoproteins.^1^ This increase in plasma TGs has traditionally been attributed to an overproduction of hepatic very low density lipoprotein (VLDL) particles accompanied by a potential decrease in TG‐rich lipoprotein particle clearance.^2^, ^3^ However, a growing body of evidence suggests that deregulated intestinal lipoprotein metabolism is a key event in the onset of dyslipidemia. Numerous studies demonstrate increased postprandial plasma TGs and apolipoprotein B_48_ (apoB_48_) levels in patients who are obese and/or have type 2 diabetes due to chylomicron overproduction.^4^ The last decade has seen extraordinary advances in our understanding of the role of abnormal intestinal lipid absorption in the development of obesity and metabolic diseases, highlighting the intestine as a gatekeeper in the flux of dietary fat into the circulation. This important function is particularly evident in mice lacking diacylglycerol acyltransferase 1 (*Dgat1*), which catalyzes the final step in TG synthesis. *Dgat1*‐deficient mice are resistant to diet‐induced obesity and liver steatosis. In contrast, the recovery of Dgat1 expression solely in the small intestine renders these mice susceptible to obesity and NAFLD. The enzymes involved in intestinal TG synthesis and chylomicron production are well established,^5^ whereas the role and nature of TG‐mobilizing enzymes in the small intestine await further examination. The catabolism of TGs from cytosolic lipid droplets (LDs) requires the TG hydrolase (TGH) activity of adipose triglyceride lipase (Atgl) and its coactivator comparative gene identification‐58 (Cgi‐58) in various tissues. Disruption of Cgi‐58 expression in the liver or intestine provokes hepatic and intestinal fat accumulation, respectively, which was less pronounced in mice lacking Atgl in these organs. Moreover, hepatic and intestinal TGH activities were only moderately reduced in these mouse models. These findings indicate the presence of other unknown TG lipases in the intestine. The search for alternative TGHs in murine CGI‐58‐deficient tissues through activity‐based protein profiling revealed a marked increase in Ces2c protein expression (unpublished). Recently, two independent studies demonstrated that Ces2c/CES2 can act as a TGH and that reduced hepatic expression of Ces2c/CES2 is linked to NAFLD development in obese mice and humans.^6^, ^7^ *Ces2c* is expressed in lipoprotein‐producing organs with highest expression levels in the duodenum.^8^ To examine the role of Ces2c in intestinal lipid metabolism, we generated and characterized mice overexpressing *Ces2c* in the intestine. We found that intestinal *Ces2c* overexpression protects mice from excessive diet‐induced weight gain and liver steatosis. Mechanistically, *Ces2c* overexpression elevates FAO in the intestine and increases chylomicron size and clearance, thereby affecting whole‐body lipid distribution during metabolic stress.

## Methods

### Cloning and Expression of *Ces2c* in COS‐7 cells

Full‐length *Ces2c* was cloned in the pFLAG‐CMV‐5.1 vector and used for transient expression in COS‐7 cells. For stable expression in COS‐7 cells, a lentivirus was generated by cloning of *Ces2c* in the pLVX‐IRES‐Puro plasmid, followed by assembly into virions in human embryonic kidney cells. Detailed information can be found in the Supporting Information.

### TGH Activity and Diglyceride Hydrolase Assays in Cell and Tissue Lysates

TGH and diglyceride hydrolase (DGH) activity assays were performed as reported with some minor modifications.^9^ Detailed information can be found in the Supporting Information.

### FA Incorporation Studies and FAO in Cell Culture

To measure *ex vivo* FA metabolism, stably *Ces2c* transduced COS‐7 cells were incubated with radiolabeled oleic acid (OA) and starved for 4 hours. FAO measurements were performed in COS‐7 cells stably overexpressing FLAG‐tagged *Ces2c* applying radiolabeled palmitic acid (PA) as described.^10^ Detailed information can be found in the Supporting Information.

### RNA and Protein Methods

Tissue RNA was extracted using Trizol reagent, and quantitative real‐time polymerase chain reaction was conducted by applying the Applied Biosystems StepOnePlus detection system (Foster City, CA). Primers are listed in Supporting Table S2.

### Western Blot Analyses

Western blot analyses were performed using either cell lysates from COS‐7 or Expi293 cells or intestinal, liver, or *Musculus (M.) quadriceps* tissue lysates from Ces2c^int^ mice according to standard procedures.

### Animals and Diet

Transgenic mice were generated expressing murine *Ces2c* complementary DNA (cDNA) under the control of the intestine‐specific 12.4‐kb villin promoter. Details are provided in the Supporting Information. Specific pathogen‐free quality of mice was confirmed according to the current Federation for Laboratory Animal Science Associations recommendations. Animals were housed in Tecniplast type 2 lang cages (Tecniplast, Hohenpeißenberg, Germany) under standard laboratory conditions (room temperature, 21 ± 1°C [mean ± SEM]; relative humidity, 45%‐65%; photoperiod, 14L:10D) and supplied with a standard laboratory chow diet (4.5% fat) or high‐fat diet (HFD) (45% fat; ssniff Spezialdiäten GmbH, Soest, Germany) and tap water *ad libitum*. Unless otherwise stated, male mice at the age of 12 to 16 weeks were examined. Hemizygote littermates were used in the studies, and mice were backcrossed at least five times from the C57BL/6N onto the C57BL/6JRj background. Tissues and organs were collected and immediately snap frozen. Genetic modification, maintenance, handling, and tissue collection from mice was approved by the Austrian Federal Ministry for Science and Research and by the ethics committee of the University of Graz and University of Veterinary Medicine Vienna.

### Plasma Chemistry

For the analysis of plasma parameters, animals were briefly anesthetized, and blood was collected from the orbital plexus. Plasma TGs, free fatty acids (FFAs), total phospholipids, and total cholesterol were determined using commercial kits (Thermo Fisher Scientific, Waltham, MA; Merck, Darmstadt, Germany; Wako Chemicals, Neuss, Germany; DiaSys Diagnostics, Holzheim, Germany; Roche Diagnostics, Rotkreuz, Germany). Blood glucose was determined using a Wellion CALLA glucometer (Med Trust, Marz, Austria), and plasma insulin levels were measured using the mouse Insulin ELISA Kit (Hölzel Diagnostika GmbH, Köln, Germany).

### Determination of Hepatic and Intestinal lipids

Hepatic lipids were extracted according to the method of Folch et al.^11^ and measured using a commercial kit (Thermo Fisher Scientific). Intestinal tissue explants were extracted according to Matyash et al.,^12^ and targeted lipidomics analysis was performed using ultraperformance liquid chromatography mass spectrometry analysis (Bruker, Billerica, MA). Detailed information can be found in the Supporting Information.

### Postprandial Lipoprotein and Chylomicron Secretion

Postprandial lipoprotein clearance and chylomicron secretion rates were determined as described.^13^ Detailed information can be found in the Supporting Information.

### Glucose Tolerance and Dietary Fat Uptake

Glucose tolerance tests were performed in 6‐hour‐fasted mice.^14^ Fat absorption along the length of the small intestine was assessed as described.^13^

### Metabolic Phenotyping

Acclimatized mice were housed in a laboratory animal monitoring system, enabling the continuous measurement of locomotor activity, oxygen consumption, and carbon dioxide elimination. Calculation of activity‐based energy expenditure (AEE) and resting metabolic rate (RMR) was performed as described by van Klinken et al.^15^ Detailed information can be found in the Supporting Information.

### Analyses of Oxygen Consumption Rates

To examine oxygen consumption rates (OCRs), high‐resolution respirometry was performed with freshly prepared intestinal whole‐tissue lysates. Detailed information can be found in the Supporting Information.

### Fat Absorption, Fecal Analysis, and Fecal Output

Fat absorption was determined by the sucrose polybehenate method as described.^16^ Fecal output was measured on 5 consecutive days. For fecal energy content measurements, feces of single‐housed, HFD‐fed mice were burned in an adiabatic oxygen bomb calorimeter.

### Statistical Analysis

Statistical significance was determined by the unpaired Student *t* test (2‐tailed) or analysis of covariance (ANCOVA). Group differences were considered significant for *P < 0.05, **P < 0.01, and ***P < 0.001.

## Results

### Ces2c Efficiently Hydrolyzes TGs and DGs and Promotes FAO

**Figure 1:**
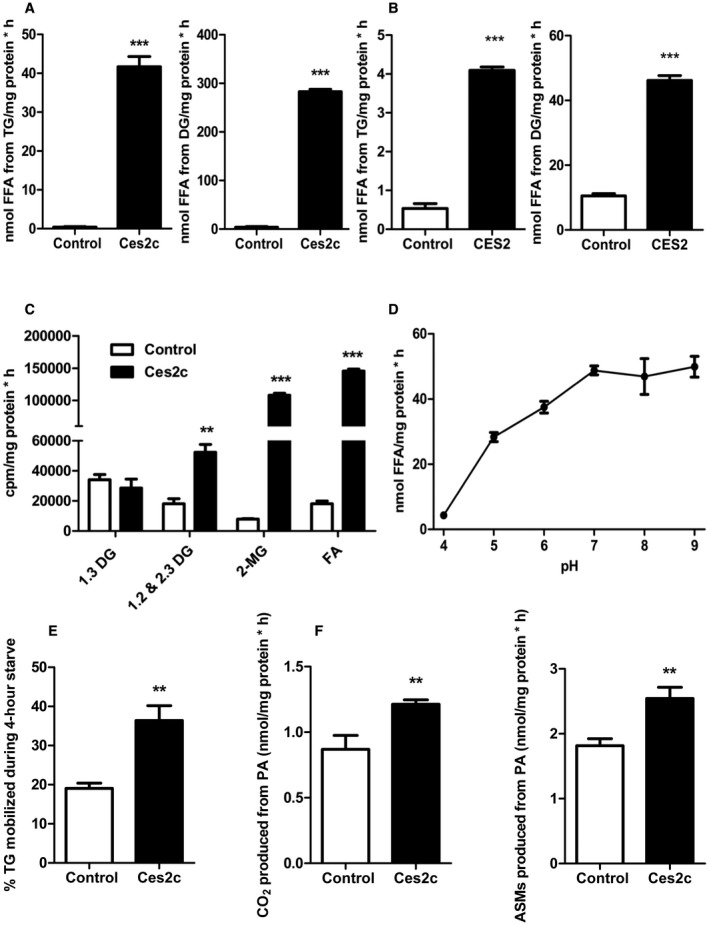
Ces2c is an efficient TGH and DGH promoting FAO. Cell lysates enriched with recombinant Ces2c (A) or CES2 (B) were incubated with a micellar [^3^H]‐labeled TG (left panel) or DG (right panel) substrate. TG/DG hydrolysis was monitored by measuring the release of FFA (n = 3). (C) TGH activity assays were performed with Ces2c‐enriched or LacZ‐enriched cell lysates followed by lipid extraction.^11^ Extracted lipids were separated by TLC, and radioactivity of DG, MG, and FA corresponding bands was determined by liquid scintillation (n = 3). (D) pH dependence of Ces2c TGH activity was measured (n = 3). (E) *Ces2c*‐overexpressing COS‐7 cells were pulse labeled with [^3^H]‐labeled OA for 20 hours and starved for 4 hours. TG levels were examined after the pulse and chase period, and percent TG mobilization was calculated (n = 6). (F) *Ces2c*‐transduced COS‐7 cells were serum‐starved overnight and incubated with [^14^C]‐PA, followed by addition of perchloric acid to release CO_2_. ASMs and the saturated filter paper containing trapped [^14^C]‐CO_2_ were assessed for radioactivity in a liquid scintillation counter (n = 6). Data represent mean + or ± SEM. Statistical significance was determined by 2‐tailed Student *t *test (**P < 0.01; ***P < 0.001). Abbreviations: cpm, counts per minute; PA, palmitic acid.

To address the role of *Ces2c* in lipid catabolism, we expressed FLAG‐tagged *Ces2c* in COS‐7 cells (Supporting Fig. S1A, left panel) and performed activity assays by applying various lipid substrates. In accordance with a previous study,^17^ Ces2c hydrolyzes palmitoylcarnitine (Supporting Fig. S1B). We also observed enzymatic activity toward para‐nitrophenyl valerate and para‐nitrophenyl acetate (Supporting Fig. S1C), which are typical esterase substrates. Two studies indicated that Ces2c also harbors TGH activity^6^, ^7^; however, enzymatic activities (levels of hydrolyzed FAs per time) were not indicated. To characterize the enzymatic function of Ces2c, we measured FA release by TGH and DGH activities in cell lysates prepared from COS‐7 cells overexpressing FLAG‐tagged *Ces2c* as described.^9^ Notably, neutral TGH and DGH activities increased 40‐fold and 70‐fold compared with control (Fig. 1A). *CES2* has been proposed as the human ortholog of murine *Ces2c*, which prompted us to measure TGH and DGH activities of CES2. As shown in Fig. 1B, TGH and DGH activities were significantly increased (8‐fold and 4‐fold, respectively) in CES2‐enriched cell preparations (Supporting Fig. S1A, right panel) compared with control. To determine whether Ces2c exhibits positional selectivity for TG hydrolysis, we incubated cytosolic fractions of Ces2c‐enriched COS‐7 cells with a [^3^H]‐labeled micellar triolein substrate and separated the products by thin layer chromatography (TLC). Ces2c‐mediated TG hydrolysis led to the formation of *stereospecific numbering* (*sn)*‐1,2 and *sn*‐2,3 DGs and *sn*‐2 monoglycerides (MGs) (Fig. 1C). Together, these findings indicate that Ces2c possesses TGH and DGH activity and has a strong positional preference for the hydrolysis of long‐chain FA esters at the *sn*‐1 and *sn*‐3 position of the glycerol backbone. Ces2c exhibited a pH optimum between 7 and 9 and lacked TGH activity under acidic conditions (Fig. 1D). To explore the role of Ces2c in intracellular TG catabolism, we used [^3^H]‐OA pulse–chase experiments to address the incorporation of radiolabeled FAs into cellular TGs and subsequent TG turnover. *Ces2c* expression in COS‐7 cells led to similar incorporation of radioactivity within TGs at the end of a 20‐hour pulse period (Supporting Fig. S1D). These findings were verified by measuring initial rates of FA uptake, which were also unaltered between *Ces2c* expressing COS‐7 and control cells (Supporting Fig. S1E). Augmented *Ces2c* expression nearly doubled TG mobilization after a 4‐hour chase period (in the absence of exogenous OA) (Fig. 1E). To determine whether *Ces2c* overexpression affects FAO, we measured the release of [^14^C]‐labeled CO_2_ and the generation of acid‐soluble metabolites (ASMs). When COS‐7 cells were incubated with [^14^C]‐PA or [^14^C]‐glucose, the overexpression of *Ces2c* resulted in an increased conversion of [^14^C]‐PA into CO_2_ and augmented the incorporation into ASMs (Fig. 1F), whereas [^14^C]‐glucose oxidation was unchanged (Supporting Fig. S1F). These data demonstrate enhanced total FAO likely due to increased FA availability from TGH/DGH activity.

### Ces2c^int^ Mice Exhibit Increased Intestinal Lipolysis and Oxidative Respiration

**Figure 2:**
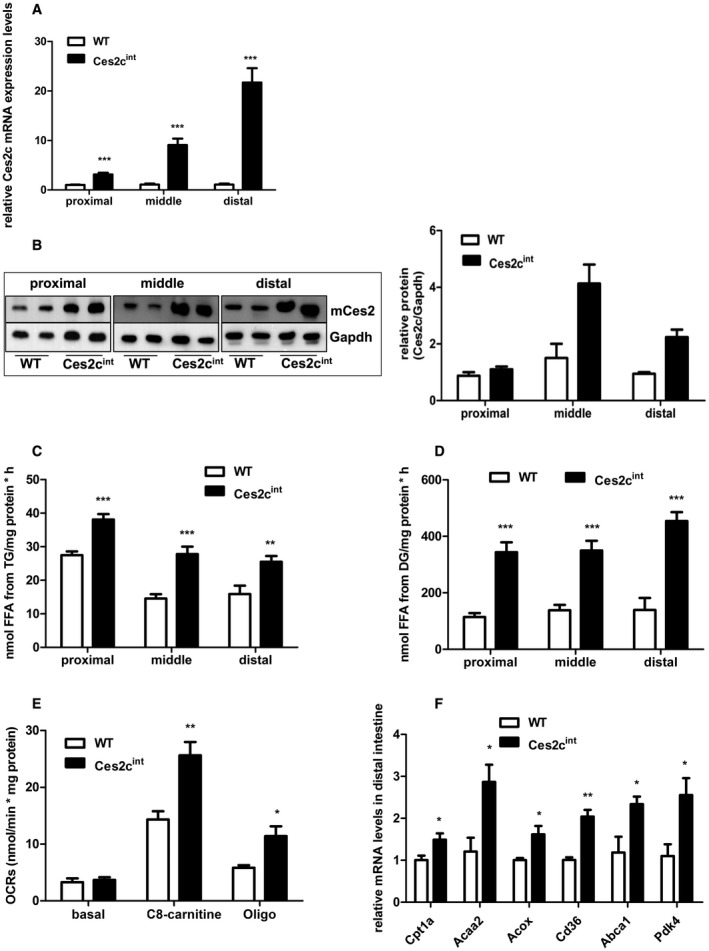
Intestine‐specific *Ces2c* overexpression increases intestinal lipolysis and respiration in mice. *Ces2c* mRNA (A) and protein (B, left panel) expression levels in the proximal, middle, and distal small intestine were examined in re‐fed transgenic mice compared with controls (n = 6 for mRNA measurements). Relative Ces2c protein levels were determined by densitometric analysis (B, right panel). Intestinal preparations of fasted mice were incubated with a micellar [^3^H]‐TG (C) or DG substrate (D), and TGH/DGH activity was measured (n = 6). (E) The OCRs of total distal small intestine lysates were measured by applying a two‐chamber oxygraph. The respiratory capacity was analyzed in the presence of adenosine diphosphate and cytochrome c by adding C8‐carnitine (FAO substrate) and oligomycin (inhibition ATP‐synthase). OCRs were calculated per milligram of tissue protein (n = 7). (F) The mRNA expression levels of Ppara target genes were examined in the small intestine of transgenic mice and compared with controls (n = 6). Data represent mean + SEM. Statistical significance was determined by 2‐tailed Student *t* test (*P < 0.05; **P < 0.01; ***P < 0.001). Abbreviations: Abca1, ATP binding cassette subfamily A member 1; Acaa2, acetyl‐CoA acyltransferase 2; Acox, acyl coenzyme A (CoA) oxidase; C8‐carnitine, octanoyl‐CoA‐L‐carnitine; CD36, cluster of differentiation 36; Ces2c, carboxylesterase 2c (murine); Cpt1a, carnitine palmitoyl‐transferase 1 alpha; Gapdh, glyceraldehyde‐3‐phosphate dehydrogenase; Oligo, oligomycin; Pdk4, pyruvate dehydrogenase kinase isoenzyme 4; WT, wild type.

To address the role of Ces2c in intestinal lipid and energy metabolism *in vivo*, we generated mice expressing FLAG‐tagged *Ces2c* cDNA under the control of the villin promoter/enhancer (Supporting Fig. S2A). Four transgenic lines were produced by pronuclear microinjection on a C57BL/6NRj background, Tg(Ces2c)754‐757Biat, hereinafter designated as Ces2c^int^. However, transgene protein expression was merely elevated in two lines, demonstrating a comparable increase in Ces2c protein levels (data not shown). For further investigation, we selected line 2 showing a 3‐fold, 9‐fold, and 22‐fold increase in *Ces2c* messenger RNA (mRNA) levels in the proximal, middle, and distal small intestine of Ces2c^int^ mice compared with littermate controls (Fig. 2A). Total Ces2c protein levels (encompassing endogenous and transgene expression) increased approximately 1.3 fold, 3.0 fold, and 2.3 fold in the proximal, middle, and distal small intestine (Fig. 2B), which is in line with increased protein levels of the *Ces2c* transgene (Supporting Fig. S2B). Next, we measured endogenous Ces2c protein levels in dependence of the nutritional state. We observed a moderate but insignificant increase in Ces2c protein levels in the middle and distal small intestine of fed wild‐type (WT) mice compared with the fasted state (Supporting Fig. S2C,D), implicating that Ces2c protein expression is not under strict nutritional regulation. In line with increased TGH/DGH activities of Ces2c‐enriched cell lysates (Fig. 1A), intestinal *Ces2c* overexpression significantly elevated lipid hydrolytic activities in the proximal, middle, and distal parts of the small intestine (up to +91% for TGH activities and up to +225% for DGH activities, respectively) compared with WT (Fig. 2C,D). High‐resolution respiratory measurements revealed a significant increase in the respiratory capacity when Octanoyl‐CoA‐L‐Carnitine was used as substrate in fresh tissue lysates prepared from the distal small intestine of Ces2c^int^ mice compared with controls (Fig. 2E), suggesting increased FAO. Additionally, respiratory capacities were moderately elevated in the proximal small intestine of Ces2c^int^ mice, whereas levels were unchanged in lysates prepared from the middle small intestine (Supporting Fig. S2E). The increase in respiratory capacity was paralleled by increased mRNA expression of FAO genes, which are under regulation of peroxisome proliferator‐activated receptor alpha (Ppara), including carnitine palmitoyl‐transferase 1a (Cpt1a), acetyl‐CoA acyltransferase 2, acyl‐CoA oxidase (Acox), cluster of differentiation 36 (CD36), adenosine triphosphate (ATP) binding cassette subfamily A member 1, and pyruvate dehydrogenase kinase‐4 (Pdk4) (Fig. 2F). Thus, having characterized TGH and DGH activity in Ces2c^int^ animals, we next aimed to examine the role of Ces2c in lipid and energy metabolism in the small intestine.

### Ces2c^int^ Mice Are Protected From HFD‐Induced Obesity

**Figure 3:**
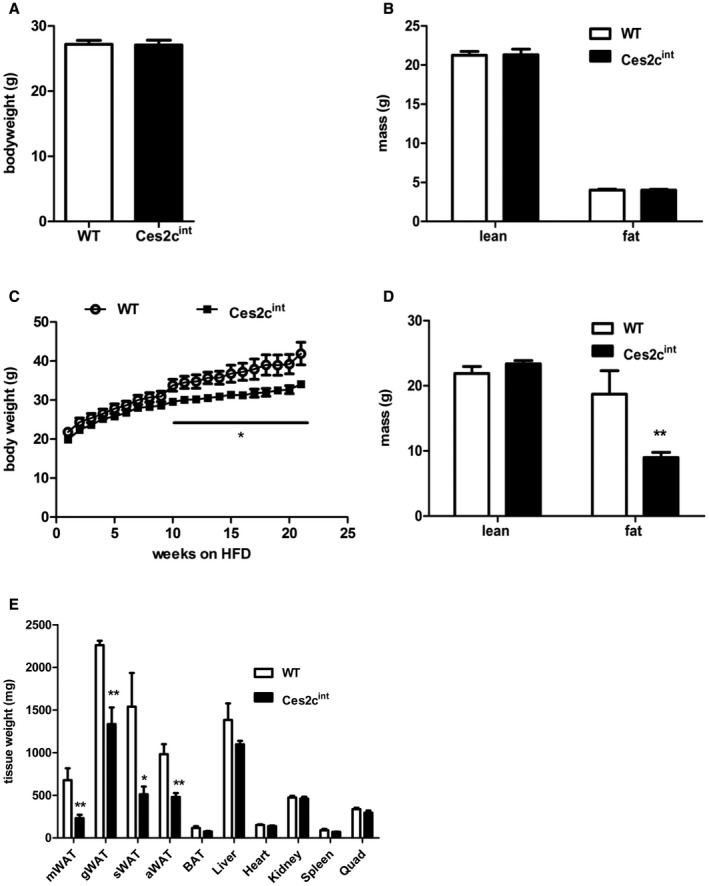
Ces2c^int^ mice are protected from HFD‐induced obesity. Body weight (A) and body composition (B) of 14‐week‐old Ces2c^int^ and control mice was determined. (C) Weight gain of 6‐week‐old male mice on HFD (45 kJ% fat; 22.1 kJ/g) was monitored for 21 weeks (n = 6). (D) Lean body mass and fat mass (indicated in grams of body weight) of 30‐week‐old Ces2c^int ^and control mice on HFD were determined with a calibrated minispec nuclear magnetic resonance analyzer (n = 6). (E) Measurements of tissue weights from 30‐week‐old Ces2c^int ^and control mice on HFD, respectively (n = 6). Data represent mean + or ± SEM. Statistical significance was determined by 2‐tailed Student *t *test (*P < 0.05; **P < 0.01). Abbreviations: aWAT, axillary white adipose tissue; BAT, brown adipose tissue; gWAT, gonadal white adipose tissue; mWAT, mesenteric white adipose tissue; Quad, quadriceps; sWAT, subcutaneous white adipose tissue.

Intestinal *Ces2c* overexpression did not affect body weight (Fig. 3A) and body mass composition (Fig. 3B) in mice fed a regular chow diet. Interestingly, intestinal Ces2c protein expression is altered in WT mice after HFD feeding (Supporting Fig. S3A). That said, we observed reduced Ces2c protein expression in the proximal small intestine of HFD‐fed WT mice, whereas protein levels were increased in the middle and proximal small intestine. In contrast, Ces2c protein expression was unchanged in the middle and distal small intestine of Ces2c^int^ mice on HFD, whereas levels were reduced in the proximal part (Supporting Fig. S3B). Moreover, *Ces2c* mRNA expression was significantly upregulated in HFD‐fed WT mice, albeit mRNA levels were highest in the middle and distal small intestine of Ces2c^int^ mice (Supporting Fig. S3C). Therefore, we examined the effect of HFD‐induced metabolic stress on weight gain in the transgenic mice. Six‐week‐old Ces2c^int^ mice were fed an HFD containing 45% calories from fat for 21 weeks. Ces2c^int^ and control mice exhibited a positive energy balance and gained weight at the same rate for the first 6 weeks. Starting from week 10, weight gain was lower in mice compared with control mice (Fig. 3C). After 21 weeks, Ces2c^int^ mice weighed approximately 25% less than controls. Nuclear magnetic resonance analysis revealed that the differences in body weight were largely due to a 50% reduction in fat mass with no changes in total lean body mass (Fig. 3D). All investigated fat depots, including mesenteric, gonadal, subcutaneous, and axonal white adipose tissue, were reduced (−64%, −41%, −61%, and −48%, respectively) in Ces2c^int^ compared with controls on HFD (Fig. 3E) but were unaltered on chow diet (Supporting Fig. S3D). These results suggest that intestine‐specific *Ces2c* expression protects mice from HFD‐induced weight gain and obesity.

### Intestinal *Ces2c* Overexpression Protects Mice From HFD‐Induced NAFLD and Improves Hepatic Insulin Sensitivity

**Figure 4:**
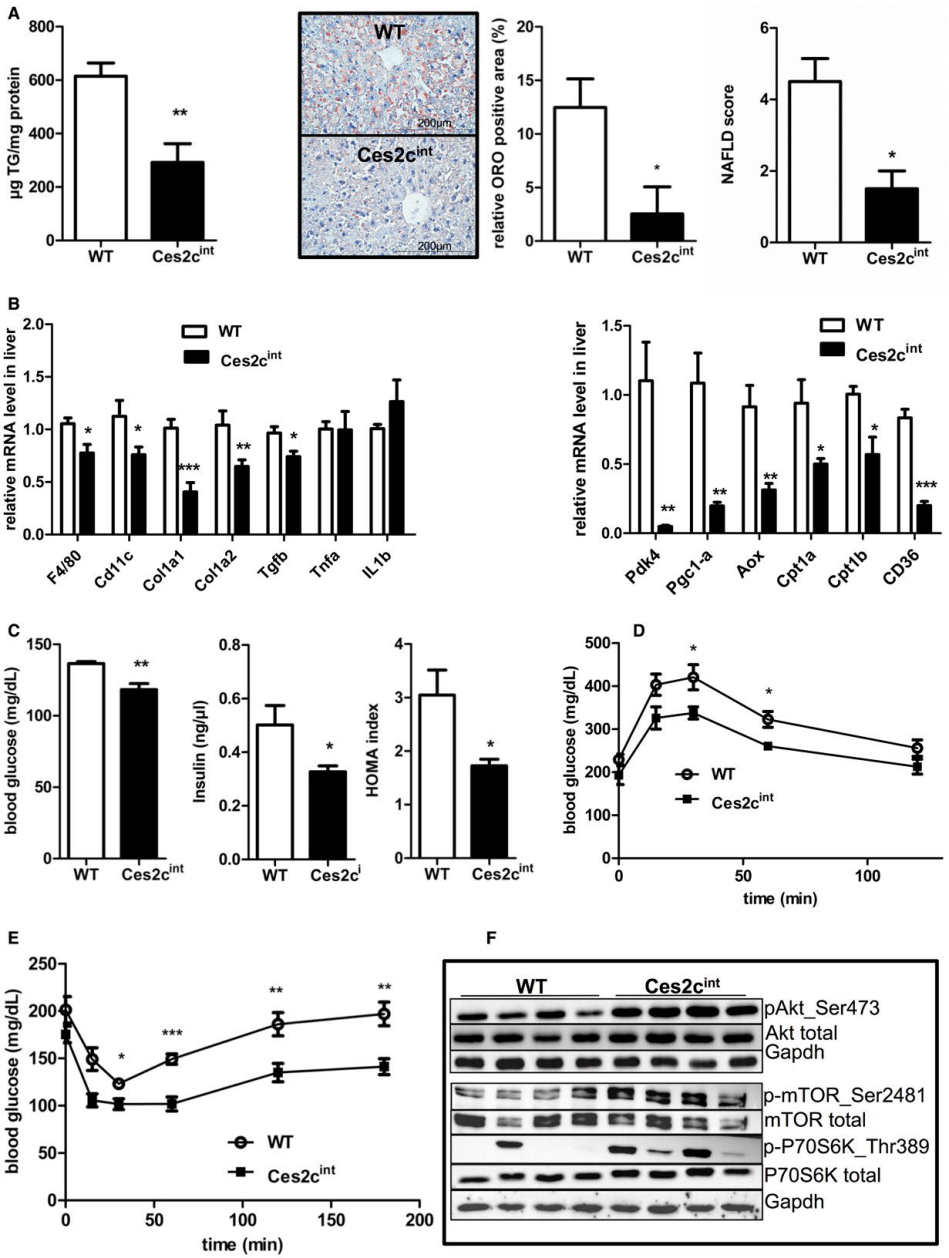
Intestinal *Ces2c* overexpression protects mice from HFD‐induced NAFLD and improves hepatic insulin sensitivity. Six‐week‐old male mice were fed an HFD (45 kJ% fat; 22.1 kJ/g) for 24 weeks. (A) Hepatic TG content (left panel) was determined (n = 5). Neutral lipids were stained by Oil Red O. Oil Red O positive particle size (area) was measured and the relative amount of positive tissue to the whole area was calculated using ImageJ with the IHC Toolbox (middle panel). Liver tissue sections were categorized according to LD abundance, inflammatory foci, and cell ballooning to determine the NAFLD score (n = 4) (right panel). (B) mRNA expression levels of inflammatory and fibrosis markers (left panel) and Ppara target genes (right panel) were examined in livers of Ces2c^int^ mice and compared with controls (n = 10; n = 6). (C) 12‐hour fasting blood glucose (left panel) and insulin (middle panel) levels were measured, and the HOMA of insulin resistance index was calculated (right panel) (n = 6). (D) Glucose tolerance test in 6‐hour‐fasted mice on HFD. (E) Insulin tolerance test in 4‐hour‐fasted mice on HFD. (F) Hepatic expression levels of proteins linked to insulin signaling determined by western blot analysis (n = 4). Data represent mean + or ± SEM. Statistical significance was determined by 2‐tailed Student *t* test (*P < 0.05; **P < 0.01; ***P < 0.001). Abbreviations: Akt, protein kinase B; Aox, acyl coenzyme A (CoA) oxidase; Cd11c, integrin alpha X; Col1a1, collagen type 1 alpha 1; Gapdh, glyceraldehyde‐3‐phosphate dehydrogenase; IL1b, interleukin 1 beta; ORO, Oil Red O; p‐Akt, phospho‐protein kinase B; mTOR, mammalian target of the rapamycin; Pgc1a, peroxisome proliferator‐activated receptor gamma coactivator 1 alpha; S6K, ribosomal protein S6 kinase beta‐1; TGF‐beta, transforming growth factor‐beta; TNFa, tumor necrosis factor‐alpha.

Next, we investigated whether intestine‐specific *Ces2c* overexpression protects mice from the development of HFD‐induced metabolic disorders such as hepatic steatosis, cholesterolemia, and impaired glucose tolerance. After long‐term HFD feeding, measurement of plasma parameters revealed a significant reduction (−20%) in total cholesterol levels in Ces2c^int ^mice, whereas FA, total phospholipid, and TG concentrations were comparable to controls (Supporting Table S1). After a 6‐hour fast, blood glucose levels tended to be lower in Ces2c^int^ mice compared with WT, although differences did not reach statistical significance. Notably, liver TG levels were substantially reduced (−52%) in Ces2c^int^ mice compared with controls (Fig. 4A, left panel), whereas the liver/body weight ratio was unchanged (Supporting Fig. S4A). In line with reduced hepatic TG levels, staining of neutral lipids through Oil Red O was markedly reduced in liver tissue sections of Ces2c^int ^compared with control mice (Fig. 4A, middle panel). Ces2c^int^ mice displayed a lower total NAFLD score as a measure of hepatic steatosis–associated histological markers (inflammation, fibrosis, and ballooning) (Fig. 4A, right panel). The reduction in hepatic TG levels on HFD prompted us examine the effect of an acute dietary fat challenge on hepatic TG homeostasis. Therefore, we performed an oral fat tolerance test (OFTT) in chow‐fed mice. Notably, hepatic TG levels were significantly lower in Ces2c^int^ mice compared with controls (Supporting Fig. S4B), further demonstrating an important role of intestinal *Ces2c* in hepatic TG homeostasis. In accordance with the lower NAFLD score on HFD, mRNA expression levels of representative macrophage/inflammatory markers (F4/80, −26%; integrin alpha X, −36%) and markers for hepatic fibrosis (collagen type I alpha 1, −50%; collagen type I alpha 2, −38%; transforming growth factor beta, −23%) were significantly decreased (Fig. 4B, left panel). Moreover, hepatic mRNA expression of genes involved in mitochondrial FA uptake and oxidation, including CD36, Pdk4, PPAR gamma coactivator‐1alpha (Pgc‐1α), Acox, and Cpt1a/b, were strongly reduced (up to −95%) in Ces2c^int^ mice compared with controls (Fig. 4B, right panel), indicating reduced hepatic FA uptake and oxidation in Ces2c^int^ mice. Hepatic mitochondrial DNA content of Ces2c^int^ mice was comparable to controls (Supporting Fig. S4C). Next, we examined the effect of HFD‐induced metabolic stress on glucose metabolism and insulin levels. Blood glucose levels were significantly reduced (−15%) in overnight‐fasted Ces2c^int^ mice compared with controls (Fig. 4C, left panel), paralleled by reduced insulin levels (Fig. 4C, middle panel). Together with low insulin levels, the significant reduction in the homeostasis model assessment (HOMA) index, as a measure of insulin resistance (Fig. 4C, right panel), suggests increased insulin sensitivity in Ces2c^int ^mice. The enhanced glucose clearance from the circulation following intraperitoneal injection of glucose (Fig. 4D) or insulin (Fig. 4E) further corroborates our assumption that intestinal *Ces2c* overexpression protects from HFD‐induced insulin resistance. Additionally, we observed increased phosphorylation of protein kinase B at Ser473, mammalian target of the rapamycin at Ser2481, and ribosomal protein S6 kinase beta‐1 at Thr389 in the liver of Ces2c^int ^mice compared with controls (Fig. 4F; Supporting Fig. S4D). In contrast, phosphorylation of the aforementioned proteins was similar in *M. quadriceps* of Ces2c^int^ mice compared with WT (Supporting Fig. S4E). In summary, these findings argue for an improvement of hepatic but not systemic insulin sensitivity and show that intestinal Ces2c overexpression protects mice from HFD‐induced NAFLD.

### Ces2c^int^ Mice Show Normal Food Intake and Mild Changes in Energy Expenditure on HFD

**Figure 5:**
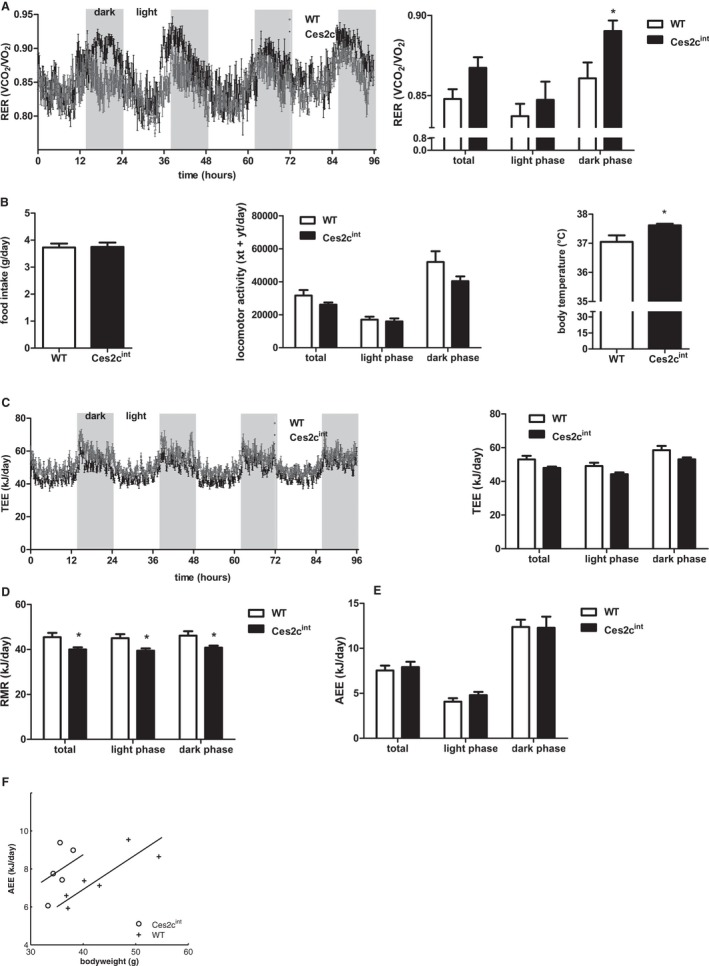
Ces2c^int^ mice show normal food intake and mild changes in energy expenditure on HFD. Six‐week‐old male mice were fed an HFD (45 kJ% fat; 22.1 kJ/g) for 24 weeks. (A) Averaged RER (VCO_2_/VO_2_) of controls and Ces2c^int^ mice over 3 days (left panel) and averaged total, light‐phase, and dark‐phase RER (right panel) (n = 5). (B) Food intake measured every second day for 2 weeks (left panel). Averaged total, light‐phase, and dark‐phase locomotor activity (middle panel) and body temperature in Ces2c^int^ and control mice (right panel) (n = 5), xt, breaks x‐beam total; yt, breaks y‐beam total. (C) TEE is displayed as averaged kJ/day over 3 days (left panel) and averaged total, light‐phase, and dark‐phase TEE (right panel) (n = 5). RMR (D) and AEE (E) were estimated as described in van Klinken et al.^15^ (F) Linear regression analysis of AEE is shown. Data represent mean + or ± SEM. Statistical significance was determined by 2‐tailed Student *t* test and ANCOVA (*P < 0.05). Abbreviation: RER, respiratory exchange ratio; VCO_2_, volume of carbon dioxide; VO_2_, volume of oxygen; xt, breaks x‐beam total; yt, breaks y‐beam total.

The leaner phenotype observed in Ces2c^int^ mice on HFD can be explained by different mechanisms, including changes in food intake and/or energy expenditure (EE). To address the consequences of intestinal *Ces2c* overexpression on whole‐body energy catabolism, we housed mice in metabolic cages. Oxygen consumption and carbon dioxide output were continuously measured to calculate respiratory exchange rate (RER). We observed no significant changes in means of total and light‐phase RER, with values at approximately 0.85 indicating the combustion of a mixture of fat and carbohydrates. Interestingly, RER was significantly increased in Ces2c^int^ mice during the dark phase, indicating a shift toward carbohydrate use as oxidative fuel (Fig. 5A). Daily food intake (Supporting Fig. S5A, left panel) or total food intake monitored over a period of 2 weeks on HFD (Fig. 5B, left panel) was essentially unchanged between Ces2c^int^ mice compared with controls. Activity levels were also comparable between groups (Fig. 5B, middle panel). Interestingly, body temperature was slightly increased in Ces2c^int^ mice (Fig. 5B, right panel). Total energy expenditure (TEE) was lower in Ces2c^int^ mice compared with controls (*P* = 0.07) (Fig. 5C), which was due to a lower RMR (*P* < 0.05) (Fig. 5D). In contrast, AEE was unchanged (Fig. 5E). Because body weight was different between both groups and body weight is an important determinant for EE, we reanalyzed EE with ANCOVA. After correction for body weight, there was no difference between groups in TEE (*P* = 0.99) and RMR (*P* = 0.44). Because heavier animals use more energy for the same amount of activity, we also assessed AEE with ANCOVA (Fig. 5F). AEE was moderately higher in Ces2c^int^ mice (*P* < 0.05) when correcting for body weight, possibly contributing to the divergence in body weight. In contrast, metabolic parameters were comparable with genotypes kept on chow diet (Supporting Fig. S5A‐F). Together, it is unlikely that the mild increase in AEE and body temperature exclusively protects mice from HFD‐induced obesity and NAFLD, respectively.

### Ces2c^int ^Mice Show Normal Lipid Absorption but Changes in Intestinal Lipid Homeostasis

**Figure 6:**
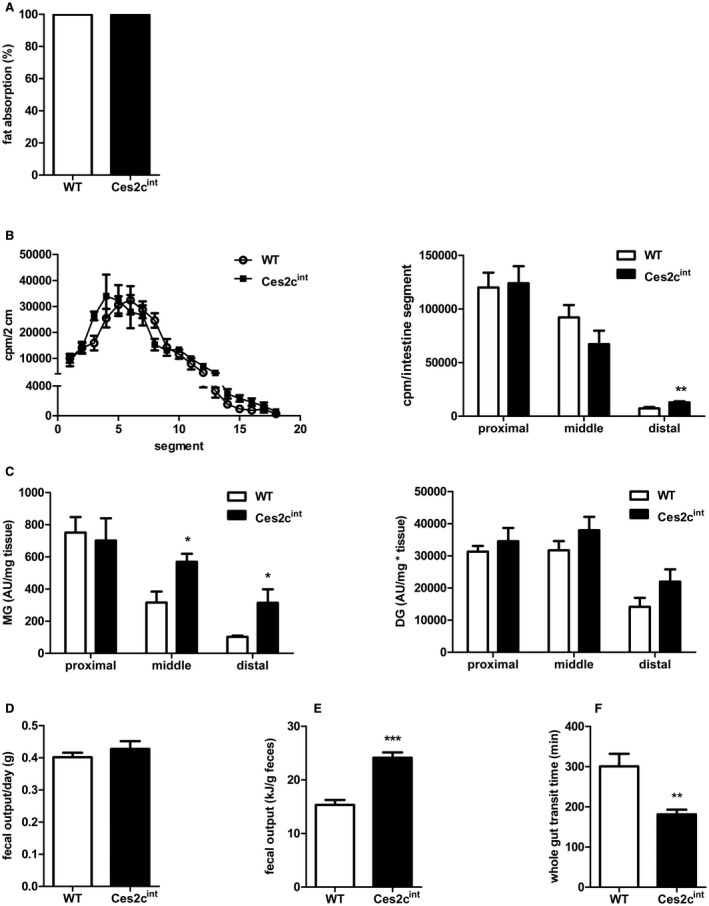
Ces2c^int^ mice show normal lipid absorption but changes in intestinal lipid homeostasis. (A) Six‐week‐old male mice were fed an HFD (45 kJ% fat; 22.1 kJ/g) for 20 weeks. Mice were individually housed and fed an HFD, including the nonabsorbable marker sucrose polybehenate (5% of the total fat), for 4 days. Lipid content and composition of the fecal pellet was determined by gas chromatography–mass spectrometry, and lipid absorption was calculated (n = 5). (B) Dietary fat uptake in the small intestine was examined in fasted chow‐fed mice after an olive oil gavage containing [^3^H]‐labeled triolein. Radioactivity was measured by liquid scintillation counting in lysed intestinal segments (left panel). Cumulative [^3^H]‐label accumulation in the proximal, middle, and distal intestine was determined (right panel) (n = 6). (C) MG (left panel) and DG levels (right panel) in the small intestine of Ces2c^int^ and control mice on HFD for 24 weeks were determined by targeted lipidomics using ultraperformance liquid chromatography mass spectrometry analysis. (D) Fecal output of 21‐week HFD‐fed mice was measured on 5 consecutive days (n = 5). (E) Feces were collected over the course of 2 weeks in Ces2c^int^ and control mice fed an HFD for 21 weeks, and fecal energy content was determined (n = 6). (F) Whole gut transit was recorded in control and Ces2c^int^ mice. Therefore, mice were gavaged with Evans blue and received free access to food. The appearance of Evans blue in the feces was recorded. Data represent mean + or ± SEM. Statistical significance was determined by 2‐tailed Student *t* test (*P < 0.05; **P < 0.01). Abbreviations: AU, Arbitrary Units; cpm, counts per minute.

Next, we determined whether differences in intestinal lipid absorption contributed to the decreased weight gain of Ces2c^int^ mice on HFD. Therefore, we fed mice an HFD that contained 5% sucrose polybehenate,^16^ collected fecal pellets, and analyzed FA content with gas chromatography mass spectrometry. Lipid absorption was essentially identical in Ces2c^int^ mice compared with controls (Fig. 6A). To confirm these data, we examined dietary fat uptake in response to an oral olive oil gavage containing [^3^H]‐labeled TGs and [^14^C]‐cholesterol as tracers. Analysis of intestinal uptake of radiolabeled TGs revealed a shift in fat absorption from the proximal to the distal intestine. Whereas uptake in the proximal intestine was similar in both genotypes, a trend toward smaller amounts of labeled lipids in the middle and a compensatory increase in fat absorption in the distal parts was observed in chow‐fed Ces2c^int^ mice (Fig. 6B). Cholesterol uptake along the small intestine was comparable in both genotypes (Supporting Fig. S6A). Next, we determined the acylglycerol levels (typically encompassing TGs, DGs, and MGs) of the proximal, middle, and distal small intestine in HFD‐fed mice. We found similar levels of acylglycerol in the proximal small intestine, whereas acylglycerol levels in the distal small intestine of Ces2c^int ^mice were significantly elevated compared with controls (Supporting Fig. S6B). This finding prompted us to analyze acylglycerol species by targeted lipidomics analysis. The measurement revealed a significant increase of MG levels in the middle and distal small intestine of Ces2c^int^ mice (Fig. 6C; Supporting Fig. S6C,D). Taken together, absorption of dietary fat is quantitatively normal in Ces2c^int^ mice. However, the spatiotemporal distribution of lipid uptake is shifted when the mice are challenged with high fat load, causing lipid accumulation in the distal parts of the small intestine of Ces2c^int^ mice. Finally, we assessed gross feces output on 5 consecutive days and found no differences in overall feces mass on HFD (Fig. 6D). However, calorimetric analysis revealed that feces from HFD‐fed Ces2c^int^ mice contained 40% more energy (kJ) than feces from controls (Fig. 6E). Calculation of average energy intake and output on HFD revealed a similar energy intake of Ces2c^int^ mice compared with controls, whereas fecal energy loss was increased (Supporting Fig. S6E). The increase in fecal energy content can be an indication for changes in whole gut transit. To address this assumption, overnight‐fasted mice received an aqueous gavage containing Evans blue, and whole gut transit time of the marker was monitored. Gut transit time was significantly decreased in HFD‐fed Ces2c^int^ mice (Fig. 6F), which may alter absorption of nonlipid metabolites and fecal energy content, respectively.

### Intestinal *Ces2c* Overexpression Increases Chylomicron Particle Size and Clearance

**Figure 7:**
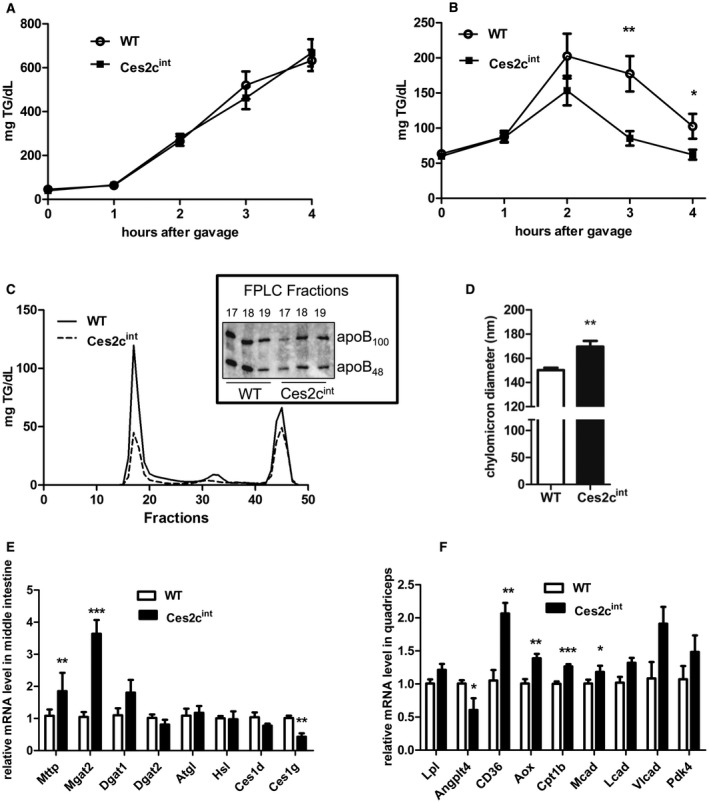
Intestinal *Ces2c* overexpression increases chylomicron particle size and clearance. (A) Measurement of plasma TG levels from fasted Ces2c^int^ and control mice over a period of 4 hours following injection of the Lpl inhibitor (tyloxapol) followed by an olive oil gavage. (B) Postprandial TG clearance of fasted Ces2c^int^ and control mice challenged with an olive oil bolus. Plasma TG levels were determined hourly (n = 6). (C) FPLC profile of the postprandial TG clearance in Ces2c^int^ and control mice 3 hours following gavage (n = 6). Western blot analysis of the lipid‐rich FPLC fractions of Ces2c^int^ and control mice (right panel). (D) The apoB_48_ lipoprotein particle size was measured by light scattering (n = 5). (E) The mRNA expression levels of genes involved in chylomicron assembly were examined in the middle small intestine of Ces2c^int^ mice and compared with controls (n = 5). (F) The mRNA expression levels of genes involved in FA uptake and oxidation in skeletal muscle. Data represent mean + or ± SEM. Statistical significance was determined by 2‐tailed Student *t *test (*P < 0.05; **P < 0.01; ***P < 0.001). Abbreviations: Ces1d, carboxylesterase 1d (murine); FA, fatty acid; Hsl, hormone sensitive lipase; Lcad, long‐chain‐acyl‐coA‐dehydrogenase; Mcad, medium‐chain‐acyl‐coA‐dehydrogenase; Mgat2, monoacylglycerol O‐Acyltransferase 2; Vlcad, very‐long‐chain‐acyl‐coA‐dehydrogenase.

Ces2c^int^ mice on HFD were protected from obesity and NAFLD development despite normal dietary fat absorption, prompting us to explore whether the entry of lipids into the circulation occurs at a reduced rate. To test this hypothesis, we performed an OFTT in the presence of the TG clearance inhibitor tyloxapol, which inhibits lipoprotein lipase (Lpl). Similar rates of TG accumulation in the plasma of chow‐fed or HFD‐fed Ces2c^int^ and control mice suggest a normal chylomicron secretion rate in Ces2c^int^ (Fig. 7A; Supporting Fig. S7A). Next, we investigated whether chylomicron clearance is affected in Ces2c^int^ mice. Therefore, mice were challenged with an olive oil gavage, and plasma TG concentrations were measured hourly up to 4 hours after the gavage. Notably, chylomicron clearance was significantly enhanced 3 and 4 hours after the gavage in chow‐fed Ces2c^int^ mice compared with controls (Fig. 7B), whereas a trend toward enhanced clearance was observed in HFD‐fed Ces2c^int^ mice (Supporting Fig. S7B). In line with the enhanced TG clearance, lipoprotein separation through fast protein liquid chromatography (FPLC) revealed a marked drop in postprandial chylomicron TG and apoB_48_ levels (Fig 7C), further suggesting increased lipoprotein TG clearance after a fat load. To address the potential mechanisms behind the efficient clearance of chylomicrons from the circulation of Ces2c^int^ mice, we isolated chylomicrons after an OFTT (in the presence of tyloxapol) and determined chylomicron size by light‐scattering measurement. Interestingly, apoB_48_ particles derived from Ces2c^int^ mice were larger in diameter (approximately 12%) compared with lipoprotein particles from controls (Fig. 7D, Supporting Fig. S7C). Additionally, mRNA expression levels of genes involved in chylomicron assembly were significantly increased in the middle part of the small intestine of Ces2c^int^ mice (Fig. 7E). Interestingly, the expression of carboxylesterase 1g (Ces1g) was significantly decreased in the middle small intestine of Ces2c^int^ mice, whereas the expression of other intestinal lipases was unaffected (Fig. 7E; Supporting Fig. S7D). Moreover, expression levels of genes involved in FA uptake and FAO in skeletal muscle were significantly elevated, accompanied by decreased expression of angiopoietin‐like 4 (Angptl4) (Fig. 7F; Supporting Fig. S7E). Finally, FA uptake into muscle tended to be increased following OFTTs, although differences did not reach statistical significance (Supporting Fig. S7F). Taken together, these results indicate that increased *Ces2c* expression in the small intestine promotes the production of larger apoB_48_ particles in mice, which are more efficiently cleared from the circulation compared with particles from WT mice.

## Discussion

Low hepatic expression of *Ces2c*/*CES2* has been linked to NAFLD development in obese mice and humans.^6^, ^7^ The aforementioned studies suggested that reduced *Ces2c/CES2* expression during metabolic stress lowers hepatic TG catabolism at the endoplasmic reticulum (ER), thereby decreasing hepatic FAO but stimulating ER stress–induced lipogenesis. Considering that *Ces2c* expression is highest in the duodenum, we hypothesized that alterations in *Ces2c* expression in the small intestine could additionally influence NAFLD development in obesity. Here we show that intestinal *Ces2c* overexpression counteracts the development of obesity and, importantly, NAFLD during HFD‐induced metabolic stress. Body weight and body mass composition of Ces2c^int^ mice were comparable to controls on chow. However, on HFD, Ces2c^int^ mice were protected from diet‐induced obesity and liver steatosis, highlighting an important function of Ces2c in the small intestine during metabolic stress, as suggested for hepatic *Ces2c*/*CES2*.^6^, ^7^ In line with the aforementioned studies exploring hepatic Ces2c function, we add additional insights into the importance of Ces2c to efficiently hydrolyze TGs and DGs, thereby generating 2‐MGs in the small intestine. Interestingly, as demonstrated by a recent study,^7^ TGH activities were manifold higher in cell lysates enriched with Ces2c (approximately 40‐fold) and CES2 (approximately 10‐fold) when compared with CES2 (approximately 2‐fold), which may be due to different assay conditions. Taken together, our study corroborates previous findings and elucidates Ces2c as a highly efficient TG‐lipase and DG‐lipase in the intestine.

How can intestine‐specific overexpression of *Ces2c* counteract the development of HFD‐induced obesity and NAFLD? The very mild changes in overall EE and normal food intake on HFD indicate other mechanism(s) protecting the mice from metabolic disease progression. Most members of the carboxylesterase gene family reside in the ER lumen,^18^ including Ces2c/CES2, which also harbors a C‐terminal ER retention signal.^19^ It has been hypothesized that lipolysis of (LDs) in the ER lumen, in addition to the microsomal triglyceride transfer protein (Mttp)–mediated lipid transfer, delivers FAs for re‐esterification and incorporation into primordial apoB_48_‐containing lipoprotein particles.^20^ In line with this hypothesis, deficiency of carboxylesterase 1d (murine) reduces VLDL particle and chylomicron production rates,^21^, ^22^ alleviating nonalcoholic steatohepatitis in mice.^23^ We hypothesized that increased lipolysis in the small intestine of Ces2c^int^ mice could equally counteract obesity and NAFLD development through changes in fat absorption and/or chylomicron production. However, although gut transit time was decreased and fecal energy content increased, neither dietary fat absorption nor chylomicron secretion rates were altered in Ces2c^int^ mice on HFD, suggesting that other mechanism(s) counteract obesity and NAFLD development. In accordance with normal TG production of *Ces2c* transgenic mice, adenovirus‐mediated *Ces2c* overexpression or knockdown did not alter hepatic VLDL particle secretion in mice.^6^ In contrast, postprandial TG clearance was enhanced in Ces2c^int^ mice, most likely due to an increase in chylomicron size, which has been demonstrated to promote TG clearance.^24^ This increase in postprandial TG clearance of Ces2c^int^ mice could be related to lipid shuttling into skeletal muscle, thereby lowering lipid flux and TG accumulation in the liver; this requires further investigation. In line with this assumption, mRNA expression of genes involved in FAO were increased in skeletal muscle but reduced in the liver of Ces2c^int^ mice. Moreover, hepatic mRNA expression of the FA transporter CD36 was reduced, indicating decreased hepatic FA flux, which might be related to increased hepatic insulin sensitivity. In contrast, skeletal muscle mRNA expression of CD36 was up‐regulated, whereas expression of Angptl4 (a known Lpl inhibitor) was decreased, further supporting the assumption that muscle FA uptake is increased. These changes might also be related to the moderate increase in AEE in Ces2c^int^ mice. Interestingly, intestinal Ces1g mRNA expression was decreased in Ces2c^int^ mice. Ces1g deficiency increases chylomicron production but leads to delayed chylomicron clearance and hepatic insulin resistance.^13^, ^25^ Although speculative, it is conceivable that Ces2c and Ces1g have a related role in intestinal lipid metabolism and that lowering Ces1g expression is an adaptation to the marked increase in *Ces2c* expression of Ces2c^int^ mice.

**Figure 8:**
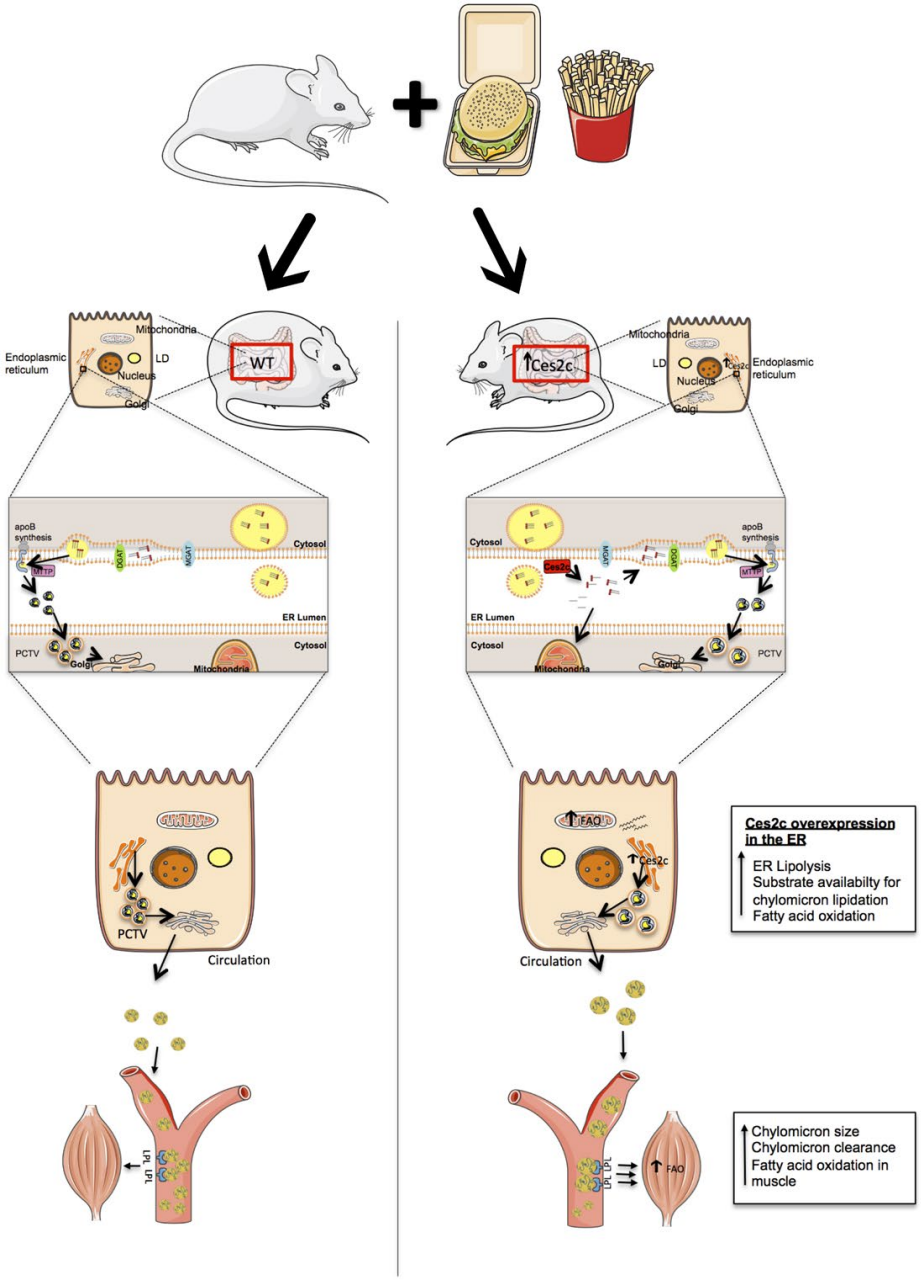
Scheme depicting the mechanisms that may protect Ces2c^int^ mice from HFD‐induced NAFLD development. Ces2c is a potent TG and DG lipase in the ER. FAs released by Ces2c promote intestinal FAO, whereas MGs and DGs are potentially used for re‐esterification and TG synthesis at the ER. As a consequence, more TGs are available for primordial apoB_48_ lipidation, leading to the generation of bigger chylomicrons. These chylomicrons are in turn more efficiently cleared from the circulation, paralleled by increased FAO in skeletal muscle. This increase in chylomicron clearance, accompanied by enhanced FAO in intestine and skeletal muscle, may counteract NAFLD and obesity development. Abbreviations: Mgat, monoacylglycerol acyltransferase; PCTV, prechylomicron transport vesicle.

Most TGs are digested to 2‐MGs and absorbed in the small intestine^26^, ^27^ for re‐esterification and generation of TGs, which are packed either in cytosolic LDs or on apoB_48_ by means of Mttp.^5^ Therefore, we propose that the increased luminal abundance of Ces2c increases TG/DG lipolysis at the ER, thereby generating FAs for oxidation and MGs/DGs for re‐esterification and TG generation, leading to the formation of more lipidated apoB_48_ particles (Fig. 8), which are efficiently cleared from the circulation. Alternatively, it is also conceivable that increased TG/DG lipolysis at the ER spatially lowers TG levels for Mttp‐mediated apoB_48_ lipidation. Nonlipidated apoB_48_ is unstable and degraded through the proteasomal pathway.^28^ Consequently, less primordial particles may acquire more TGs (and cholesterol esters), leading to an increase in particle size and efficient clearance from the circulation.

Together, this study elucidates intestinal *Ces2c*/*CES2* as a target to counteract NAFLD and obesity development.

## Potential conflict of interest

Dr. Lackner received grants from Galmed.

Author names in bold designate shared co‐first authorship.
