# Supplementary material for: Intestine‐Specific Overexpression of Carboxylesterase 2c Protects Mice From Diet‐Induced Liver Steatosis and Obesity
Source: Hepatol Commun. 2018 Dec 17;3(2):227–45. doi: 10.1002/hep4.1292 (PMC6357831; doi:10.1002/hep4.1292)
Supplement: Supplementary file 1 [file HEP4-3-227-s001.docx]

SUPPORTING ONLINE MATERIAL

**Intestine-specific overexpression of Carboxylesterase 2c protects mice from diet induced liver steatosis and obesity**

Lisa Katharina Maresch^1^, Pia Benedikt^1^, Ursula Feiler^1^, Sandra Eder^1^, Kathrin A. Zierler^1^,

Ulrike Taschler^1^, Stephanie Kolleritsch^1^, Thomas O. Eichmann^1^, Gabriele Schoiswohl^1^,
Christina Leopold^2^, Beatrix Wieser^3^, Caroline Lackner^3^, Thomas Rülicke^4^, Jan van Klinken^5^,
Dagmar Kratky^2^, Tarek Moustafa^6^, Gerald Hoefler^2^, and Guenter Haemmerle^1^

^1^Institute of Molecular Biosciences, University of Graz, Heinrichstrasse 31, 8010 Graz, Austria

^2^Gottfried Schatz Research Center, Molecular Biology and Biochemistry, Medical University of Graz, Neue Stiftingtalstraße 6, 8010 Graz, Austria

^3^Diagnostic & Research Center for Molecular BioMedicine Institute of Pathology, Medical University of Graz, Neue Stiftingtalstraße 6, 8010 Graz, Austria

^4^Institute of Laboratory Animal Science, University of Veterinary Medicine, Veterinärplatz 1, 1210 Wien, Austria

^5^Department of Human Genetics, Leiden University Medical Centre, Albinusdreef 2, 2333 ZA Leiden, the Netherlands

^6^Division of Gastroenterology and Hepatology, Medical University Graz, Auenbruggerplatz 15, 8036 Graz, Austria

The authors declare no conflict of interest.

**Supporting Tables**

**Supporting TABLE 1. Plasma parameter of Ces2c^int^ mice on HFD**

|  | **WT** | **Ces2c** |
| --- | --- | --- |
| **FFA (mmol/L)** | 0.71 ± 0.05 | 0.75 ± 0.08 |
| **TG (mmol/L)** | 0.44 ± 0.05 | 0.59 ± 1.2 |
| **CE (mg/dL)** | 243 ± 17.7 | 194 ± 27.9 |
| **total phospholipids (mmol/L)** | 4.7 ± 0.5 | 4.7 ± 0.7 |
| **Glucose (6-hour fasted)** | 230 ± 21.4 | 193 ± 43.7 |

**Supporting TABLE 2. Primer sequences used for RT-PCR**

| **Target Genes** | **Primer Sequence** |
| --- | --- |
| Abca1-fw | 5'-CTCTTCATGACTCTAGCCTGGA-3' |
| Abca1-rev | 5'-ACACAGACAGGAAGACGAACAC-3' |
| Acaa2-fw | 5'-GGACTTCTCTGCCACCGATT-3' |
| Acaa2-rev | 5'-AGAGCCACAGAGCCTGTTGA-3' |
| Angptl4-fw | 5′-GTTTGCAGACTCAGCTCAAGG-3′ |
| Angptl4-rev | 5′-CCAAGAGGTCTATCTGGCTCTG-3′ |
| Aox1-fw | 5′-AGATTGGTAGAAATTGCTGCAAAA-3′ |
| Aox1-rev | 5′-ACGCCACTTCCTTGCTCTTC-3′ |
| Apob-fw | 5′-CTGAACATCAAGAGGGGCATC-3′ |
| Apob-rev | 5′-GGTAACCTGAGTTGAGCAGTTT-3′ |
| Atgl-fw | 5’-GAGACCAAGTGGAACATC-3’ |
| Atgl-rev | 5’-GTAGATGTGAGTGGCGTT-3’ |
| Cd11c-fw | 5′-CAGTGACCCCGATCACTCTT-3′ |
| Cd11c-rev | 5′-CACCACCAGGGTCTTCAAGT-3′ |
| CD36-fwd | 5′-GAACCTATTGAAGGCTTACATCC-3′ |
| CD36-rev | 5′-CCCAGTCACTTGTGTTTTGAAC-3′ |
| Ces1d-fw | 5’-ATATGGCTTTCTCTTGCTGCG-3’ |
| Ces1d-rev | 5’-CCCAGGACTTTGCCTTTAACAGT-3’ |
| Ces1g-fw | 5’-CGAGTCAGCAGGAGGTGAAAGT-3’ |
| Ces1g-rev | 5’-TTGAAAATGACACTACTCTGAGCG-3’ |
| Ces2c-fw | 5′-GCTGAATGCTGGGTTCTTCG-3′ |
| Ces2c-rev | 5′-GCTGCCTTGGATCTGTCCTGT-3′ |
| Col1a1-fw | 5′-CCGGCTCCTGCTCCTCCTA-3′ |
| Col1a1-rev | 5′-CCATTGTGTATGCAGCTGACTTC-3′ |
| Col1a2-fw | 5′-AAGGGTGCTACTGGACTCCC-3′ |
| Col1a2-rev | 5′-TTGTTACCGGATTCTCCTTTGG-3′ |
| Cpt1a-fw | 5′-CACCAACGGGCTCATCTTCTA-3′ |
| Cpt1a-rev | 5′-CAAAATGACCTAGCCTTCTATCGA A-3′ |
| Cpt1b-fw | 5′-CGAGGATTCTCTGGAACTGC-3′ |
| Cpt1b-rev | 5′-GGTCGCTTCTTCAAGGTCTG-3′ |
| Dgat1-fw | 5′-GTGCCATCGTCTGCAAGATTC-3′ |
| Dgat1-rev | 5′-GCATCACCACACACCAATTCAG-3′ |
| Dgat2-fw | 5′-TTCCTGGCATAAGGCCCTATT-3′ |
| Dgat2-rev | 5′-AGTCTATGGTGTCTCGGTTGAC-3′ |
| F4/80-fw | 5′-GGATGTACAGATGGGGGATG-3′ |
| F4/80-rev | 5′-CATAAGCTGGGCAAGTGGTA-3′ |
| Hsl-fw | 5'-GCTGGGCTGTCAAGCACTGT-3' |
| Hsl-rev | 5'-GTAACTGGGTAGGCTGCCAT-3' |
| Il1b-fw | 5′-CACAGCAGCACATCAACAAG-3′ |
| Il1b-rev | 5′-GTGCTCATGTCCTCATCCTG-3′ |
| Lcad-fw | 5′-TTTCCGGGAGAGTGTAAGGA-3′ |
| Lcad-rev | 5′- ACTTCTCCAGCTTTCTCCCA-3′ |
| Lpl-fw | 5′-TCCAGCCAGGATGCAACA-3′ |
| Lpl-rev | 5′-CCACGTCTCCGAGTCCTCTCT-3′ |
| Mcad-fw | 5′- CAACACTCGAAAGCGGCTCA-3′ |
| Mcad-rev | 5′-ACTTGCGGGCAGTTGCTTG-3′ |
| Mgat2-fw | 5′-TGGGAGCGCAGGTTACAGA-3′ |
| Mgat2-rev | 5′-CAGGTGGCATACAGGACAGA-3′ |
| Mt-CO1-fw | 5′-TGCTAGCCGCAGGCATTAC-3′ |
| Mt-CO1-rev | 5′-GGGTGCCCAAAGAATCAGAAC-3′ |
| Mttp-fw | 5′-AGCCAGTGGGCATAGAAAATC -3′ |
| Mttp-rev | 5′-GGTCACTTTACAATCCCCAGAG -3′ |
| Ndufv1-fw | 5′-CTTCCCCACTGGCCTCAAG-3′ |
| Ndufv1-rev | 5′-CCAAAACCCAGTGATCCAGC-3′ |
| Pdk4-fw | 5′-ATCTAACATCGCCAGAATTAAACC-3′ |
| Pdk4-rev | 5′-GGAACGTACACAATGTGGATTG-3′ |
| Pgc1a-fw | 5′-CCCTGCCATTGTTAAGACC-3′ |
| Pgc1a-rev | 5′-TGCTGCTGTTCCTGTTTTC-3′ |
| Tgfb-fw | 5′-CACCGGAGAGCCCTGGATA-3′ |
| Tgfb-rev | 5′-TGTACAGCTGCCGCACACA-3′ |
| Tnfa-fw | 5′-GACCCTCACACTCAGATCATCTTCT-3′ |
| Tnfa-rev | 5′-CCTCCACTTGGTGGTTTGCT-3′ |
| Vlcad-fw | 5′-CCGGTTCTTTGAGGAAGTGAA-3′ |
| Vlcad-rev | 5′-AGTGTCGTCCTCCACCTTCTC-3′ |

**Supporting Figure Legends**

**
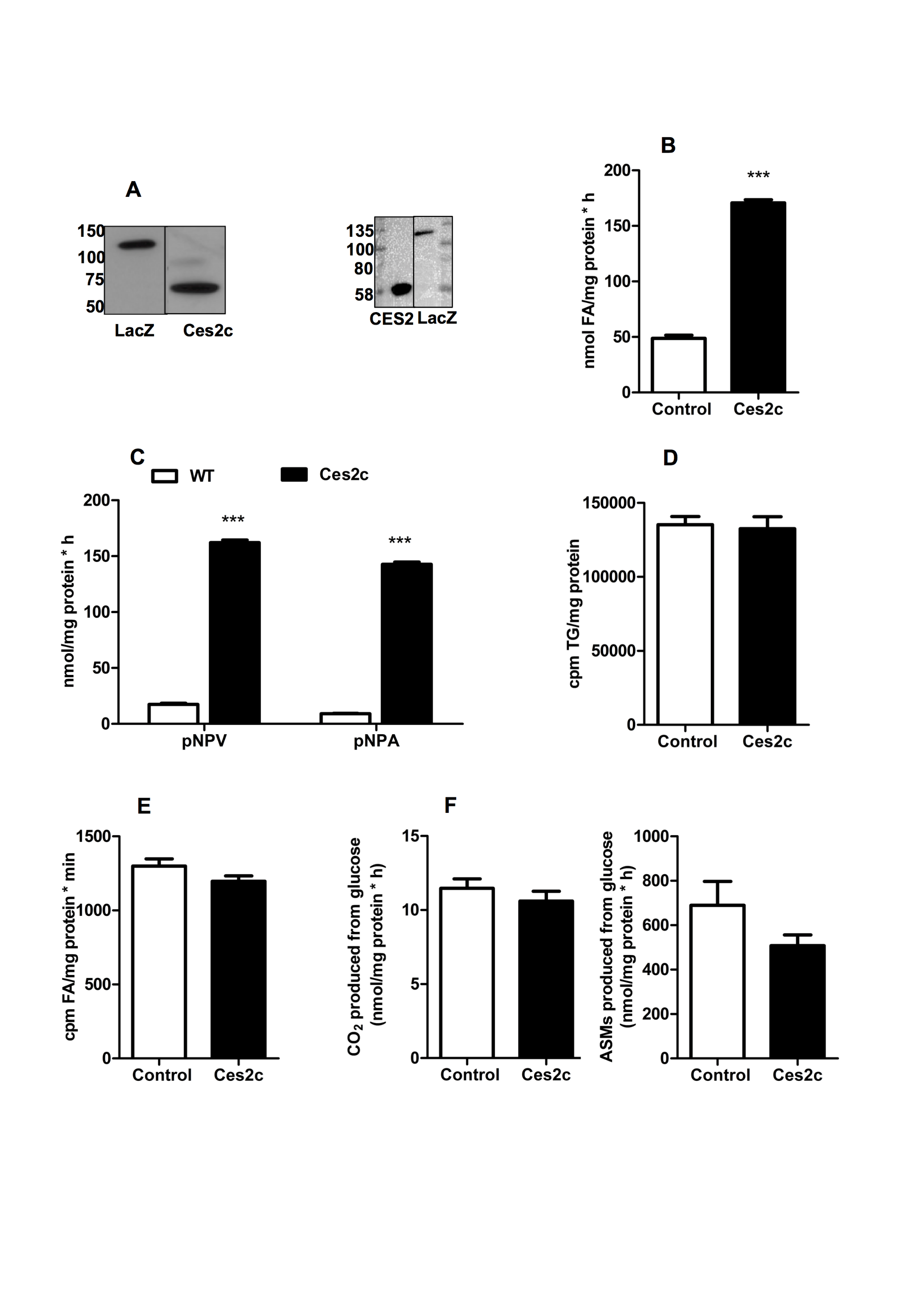
**

**Supporting FIG. 1. Enzymatic activities of Ces2c.** (A) Western blot analysis of Ces2c expression in COS-7 cells (left panel) and CES2 expression in Expi293 cells (right panel). Cell lysates containing recombinant Ces2c or lacZ/HisMax (negative control) were incubated with palmitoyl-carnitine (B), pNPV (C, left panel) or pNPA (C, right panel) (n = 3). FFA release from palmitoyl-carnitine was quantified using a commercially available kit. The rate of pNPV and pNPA hydrolysis was determined by measuring the absorbance of p-nitrophenol at 405 nm. (D) Stably Ces2c transduced COS-7 cells were incubated with [^3^H]-labeled OA for 20 hours and chased for 4 hours. Cells stably overexpressing gfp were used as a negative control. Natural lipids were extracted and separated by TLC (hexane/diethyl ether/acetic acid [70:29:1]). TG bands were cut out and quantified by liquid scintillation counting (n = 6). (E) [^3^H]-OA uptake in stably Ces2c transduced COS-7 cells (n = 6). (F) Ces2c transduced COS-7 cells were starved for 2 hours and incubated with [^14^C]-glucose, followed by addition of perchloric acid to release CO_2_. Media ASMs and the saturated filter paper containing trapped [^14^C]-CO_2_ were assessed for radioactivity in a liquid scintillation counter (n = 6). Data represent mean + SEM. Statistical significance was determined by student 2-tailed *t* test (****P* < 0.001). Abbreviation: ASMs, acid soluble metabolites; cpm, counts per minute; FFA, free fatty acids; OA, oleic acid; pNPV, para-nitrophenylvalerate; pNPA, para-nitrophenylacetate; TLC, thin layer chromatography; TG, triglycerides.

**
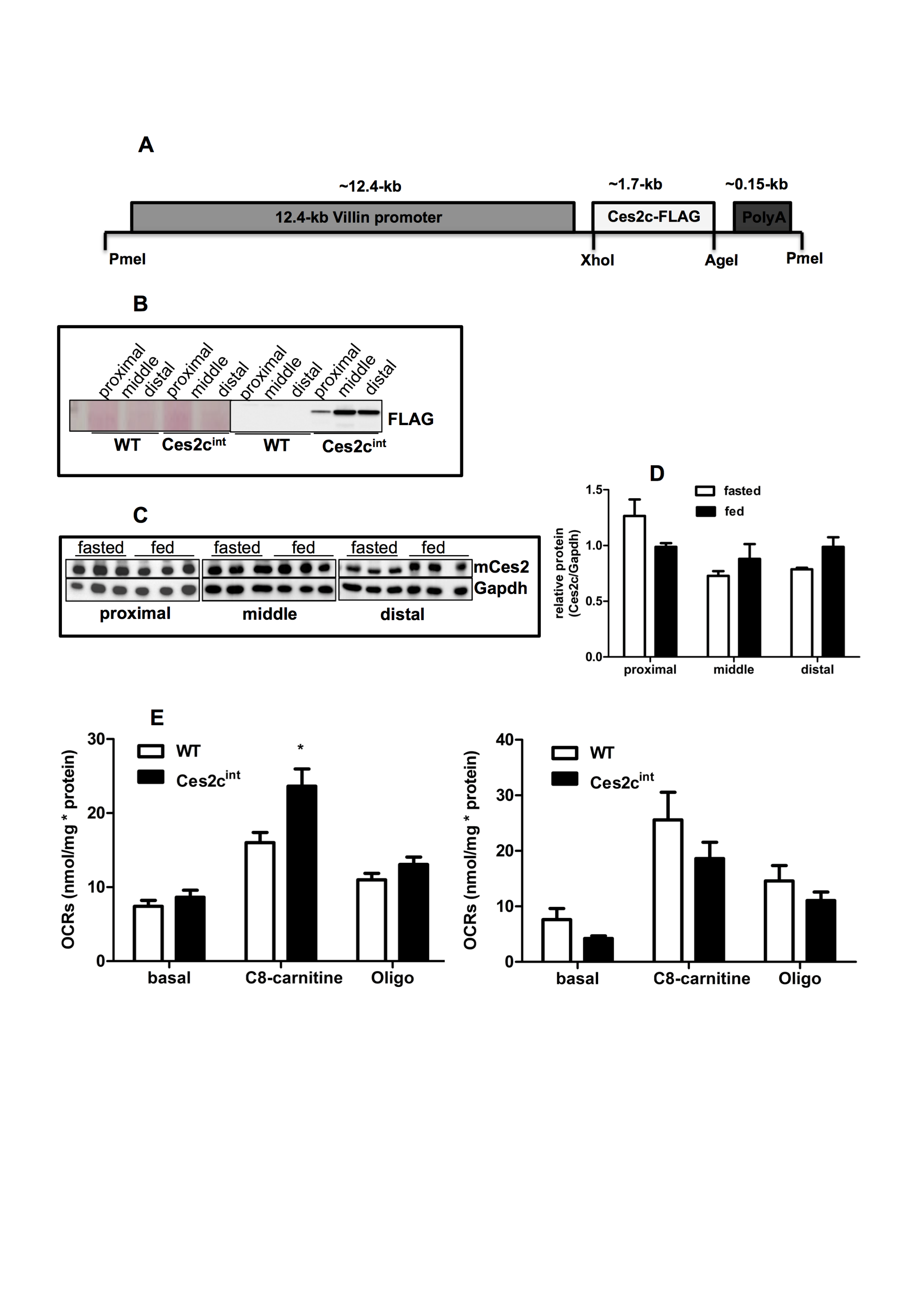
**

**Supporting FIG. 2. FAO is slightly increased in the proximal and unchanged in the middle intestine of Ces2c^int^ mice.** (A) A scheme depicting the Ces2c transgene used for the generation of transgenic mice. The C-terminal FLAG-tagged Ces2c CDS was cloned downstream of the 12.4-kb villin promoter to drive intestinal-specific overexpression of the transgene. (B) Intestinal protein expression levels of the transgene in fed Ces2c^int^ mice applying a FLAG-tag specific antibody. (C) Protein levels and densitometric analysis (D) of Ces2c levels in proximal, middle and distal small intestine of fasted or re-fed WT mice was examined. (E) OCRs of proximal (left panel) and middle small intestine lysates (right panel) were measured in a two-chamber oxygraph. The respiratory capacity was analyzed in the presence of ADP and cytochromC by adding C8-carnitine (FAO substrate) and oligomycin (inhibition ATP-synthase). OCRs were calculated per milligram of tissue protein (n = 6). Data represent mean + SEM. Statistical significance was determined by student 2-tailed *t* test (**P* < 0.05; ***P* < 0.01; ****P* < 0.001). Abbreviation: PmeI, XhoI, AgeI, restriction enzyme sites; ADP, adenosine diphosphate; C-8 carnitine, Octanoyl-CoA-L-Carnitine; CDS, coding region; Gapdh, glyceraldehyde-3-phosphate dehydrogenase; OCRs, oxygen consumption rates; Oligo, Oligomycin.

**
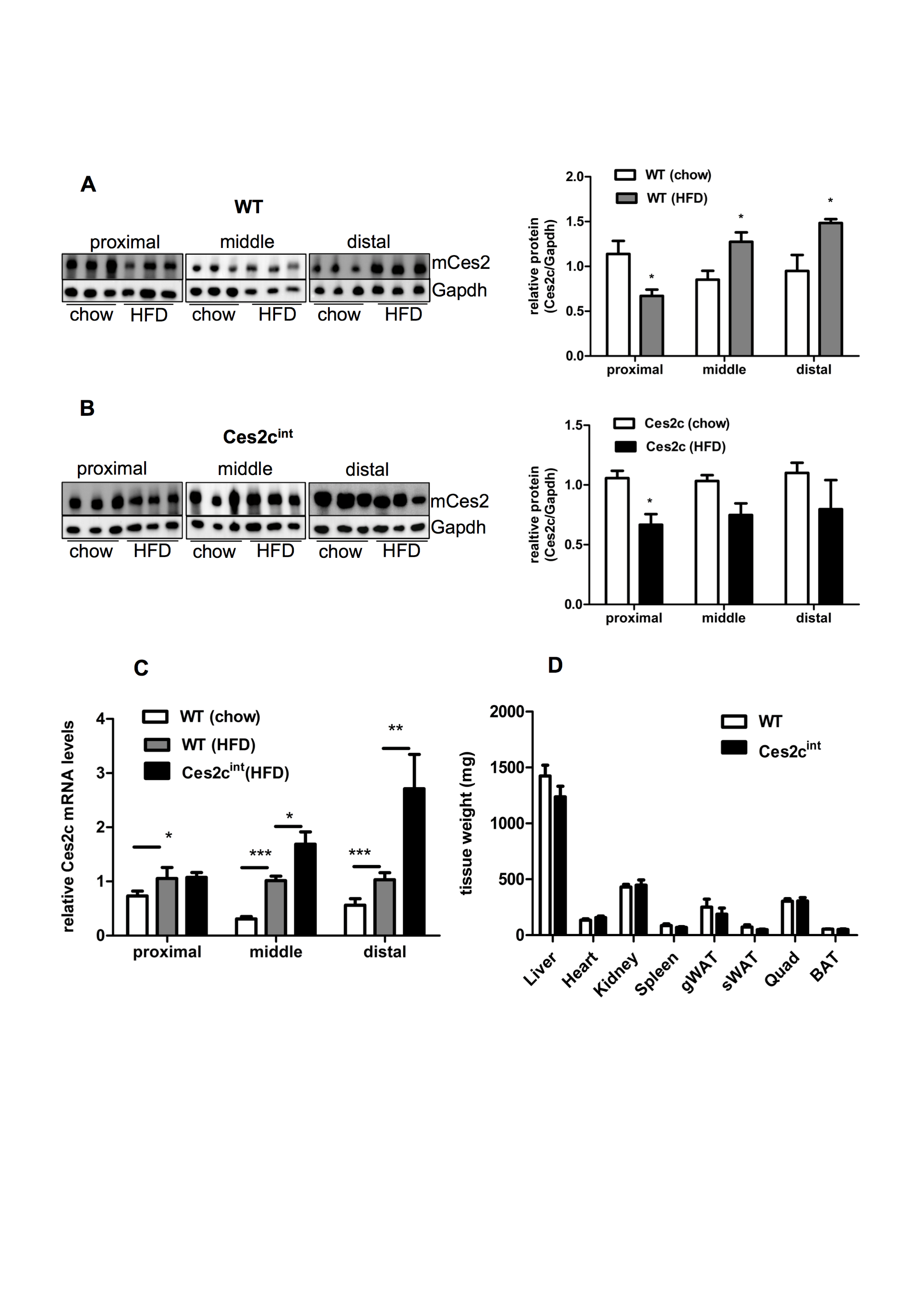
Supporting FIG. 3. Fat depot size is unaltered in Ces2c^int^ mice compared to WT mice on chow.** (A) Protein levels (left panel) and densitometric analysis (right panel) of Ces2c signal intensities in proximal, middle and distal small intestine of chow-fed or HFD-fed WT mice were examined. (B) Protein levels (left panel) and densitometric analysis (right panel) of Ces2c levels in proximal, middle and distal small intestine of chow-fed or HFD-fed Ces2c^int^ mice were compared. (C) Relative Ces2c mRNA expression levels in chow-fed or HFD-fed WT or Ces2c^int^ mice were measured. (D) Tissue weights of 14-week-old, chow-fed mice were analyzed (n = 5). Data represent mean + SEM. Statistical significance was determined by student 2-tailed *t* test (**P* < 0.05; ***P* < 0.01; ****P* < 0.001). Abbreviation: BAT, brown adipose tissue; Gapdh, glyceraldehyde-3-phosphate dehydrogenase; gWAT, gonadal white adipose tissue; HFD, high fat diet; Quad, Quadriceps; sWAT, subcutaneous white adipose tissue.


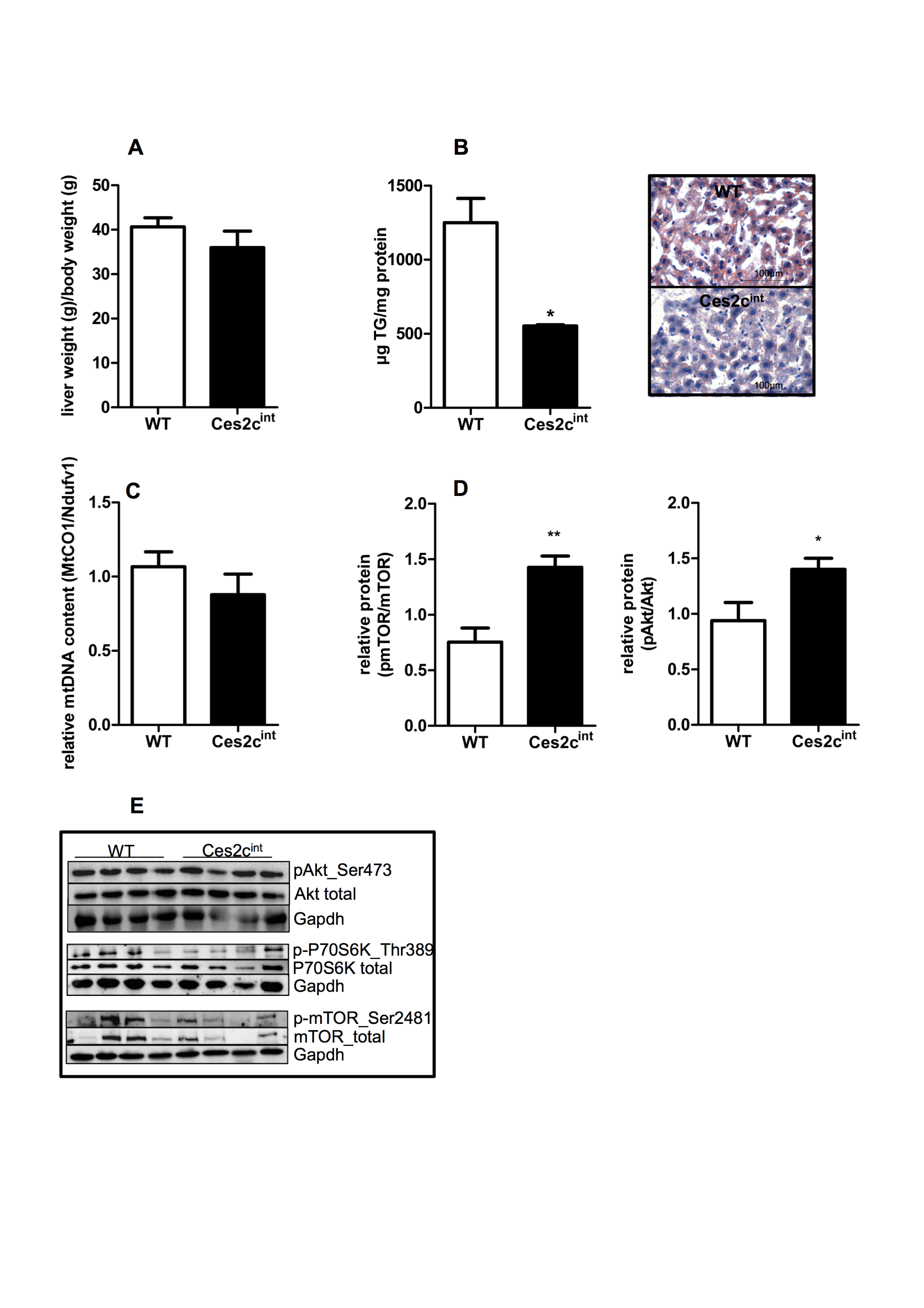


**Supporting FIG. 4. Skeletal muscle insulin signaling is comparable between Ces2c^int^ mice and WT on HFD.** (A) Analysis of the liver weight/body weight ratio of Ces2c^int^ mice on HFD (45 kJ% fat; 22.1 kJ/g) for 24 weeks. (B) OFTT was performed with 14-week-old chow fed mice. After 3 hours, livers were excised, TG content (left panel) was measured and neutral lipids were stained by Oil Red O (right panel) (n=3). (C) Relative mtDNA content in liver calculated from mRNA expression data of the mtDNA-encoded MtCO1 gene and the nuclear DNA-encoded Ndufv1 gene (n = 5). (D) Densitometric analysis of signal intensities of the western blot displayed in Fig. 4F, where protein expression in liver tissue was determined. (E) Western blot analysis of proteins involved in insulin signaling in *M. quadriceps* preparations from mice on HFD for 24 weeks (n = 4). Data represent mean + SEM. Statistical significance was determined by student 2-tailed *t* test (**P* < 0.05; ***P* < 0.01; ****P* < 0.001). Abbreviation: Akt, protein kinase B; Gapdh, glyceraldehyde-3-phosphate dehydrogenase; HFD, high fat diet; mtDNA, mitochondrial DNA; mTOR, mammalian target of the rapamycin; S6K, ribosomal protein S6 kinase beta-1.

**Supporting FIG. 5. Metabolic phenotyping of Ces2c^int^ mice on chow.** (A) Averaged total, light-, and dark-phase food intake of Ces2c^int^ mice on an HFD (left panel) or chow-diet was measured (right panel) (n = 5). Prior to metabolic cage housing, 6-month-old, chow-fed mice were familiarized to single housing for a week. (B) Averaged total, light-, and dark-phase RER is displayed (n = 5). (C) TEE was calculated and is displayed as averaged total, light‑, and dark-phase TEE (n = 5). (D) RMR and AEE (E) were estimated as described in Van Klinken et al.^(1)^ (F) Averaged total, light-, and dark-phase locomotor activity was determined (left panel) (n = 5). Body temperature of Ces2c^int^ and WT mice was measured (right panel) (n = 5). Data represent mean + SEM. Statistical significance was determined by student 2-tailed *t* test (**P* < 0.05; ***P* < 0.01; ****P* < 0.001). Abbreviation: AEE, activty based energy expenditure; RER, respiratory exchange ratio; RMR, resting metabolic rate; TEE, total energy expenditure; VCO_2_, volume CO_2_; VO_2_, volume O_2_; xt, breaks X-beam total; yt, breaks Y-beam total.

**
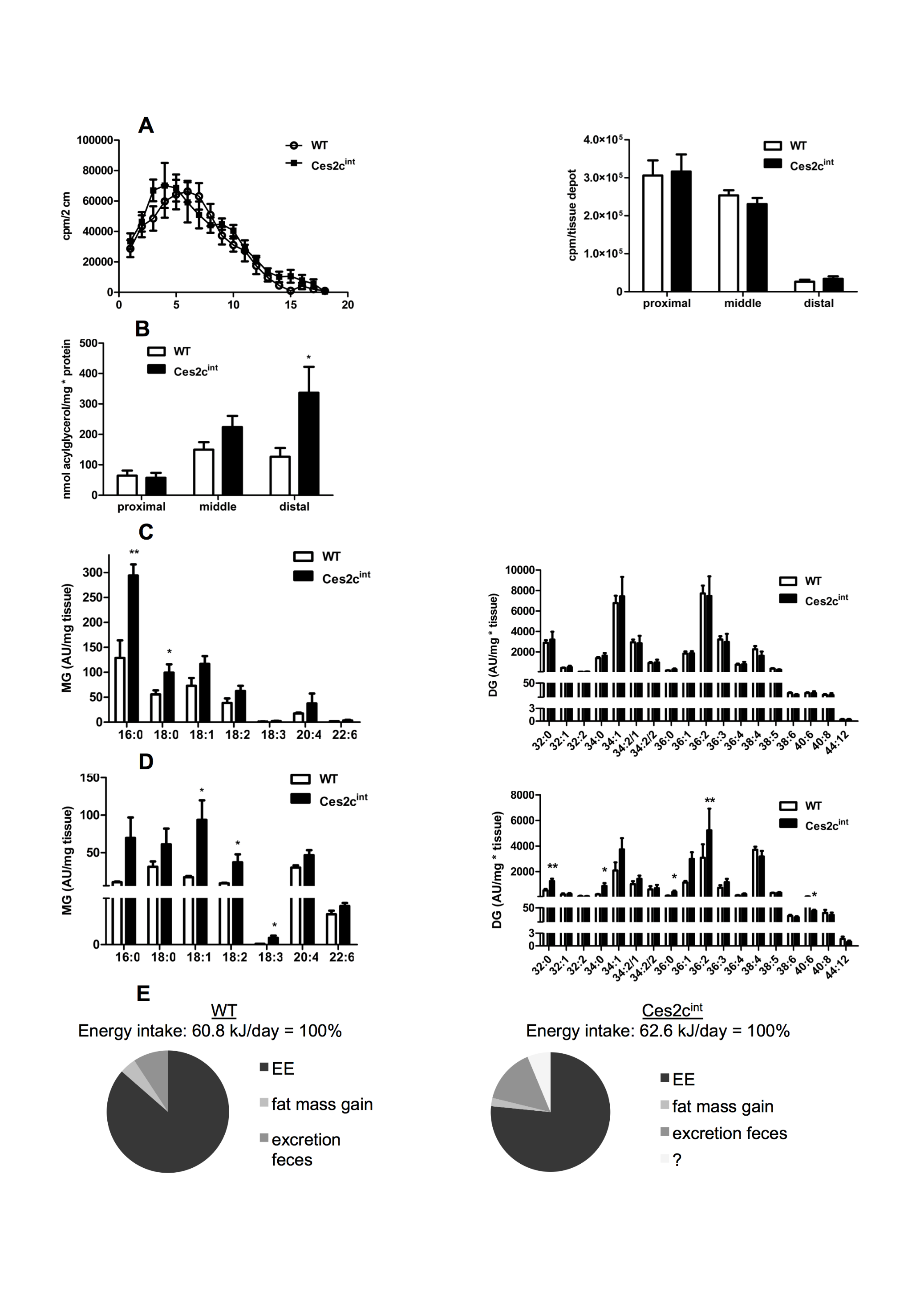
**

**Supporting FIG. 6. MG and DG species accumulation in the middle and distal small intestine of Ces2c^int^ mice on HFD.** (A) Dietary [^14^C]-cholesterol uptake in the small intestine examined in 14-week-old, chow-fed mice. Mice were fasted overnight, gavaged with olive oil containing [^14^C]-cholesterol, and sacrificed after 3 hours. The small intestine was excised, rinsed, and cut into 2-cm pieces (left panel). Radioactivity measured by liquid scintillation counting in lysed intestinal segments. Cumulative [^14^C]-label accumulation in the proximal, middle, and distal small intestine was calculated (right panel) (n = 6). (B) Measurement of acylglycerol levels in the small intestine of mice on HFD for 24 weeks (n = 5). FA species in MG (left panel) and DG levels (right panel) in the middle (C) and in the distal (D) small intestine of Ces2c^int^ and control mice on HFD for 24 weeks determined by targeted lipidomics using UPLC-MS analysis. (E) Energy balance calculated as percent of averaged energy intake (kJ/day), fecal excretion, EE, and fat mass gain per day of Ces2c^int^ mice on HFD for 24 weeks. Data represent mean + or ± SEM. Statistical significance was determined by student 2-tailed *t* test (**P* < 0.05; ***P* < 0.01; ****P* < 0.001). Abbreviation: AU, arbitrary unit; cpm, counts per minute; DG, diglycerides; EE, energy expenditure; HFD, high fat diet; MG, monoglycerides.

**
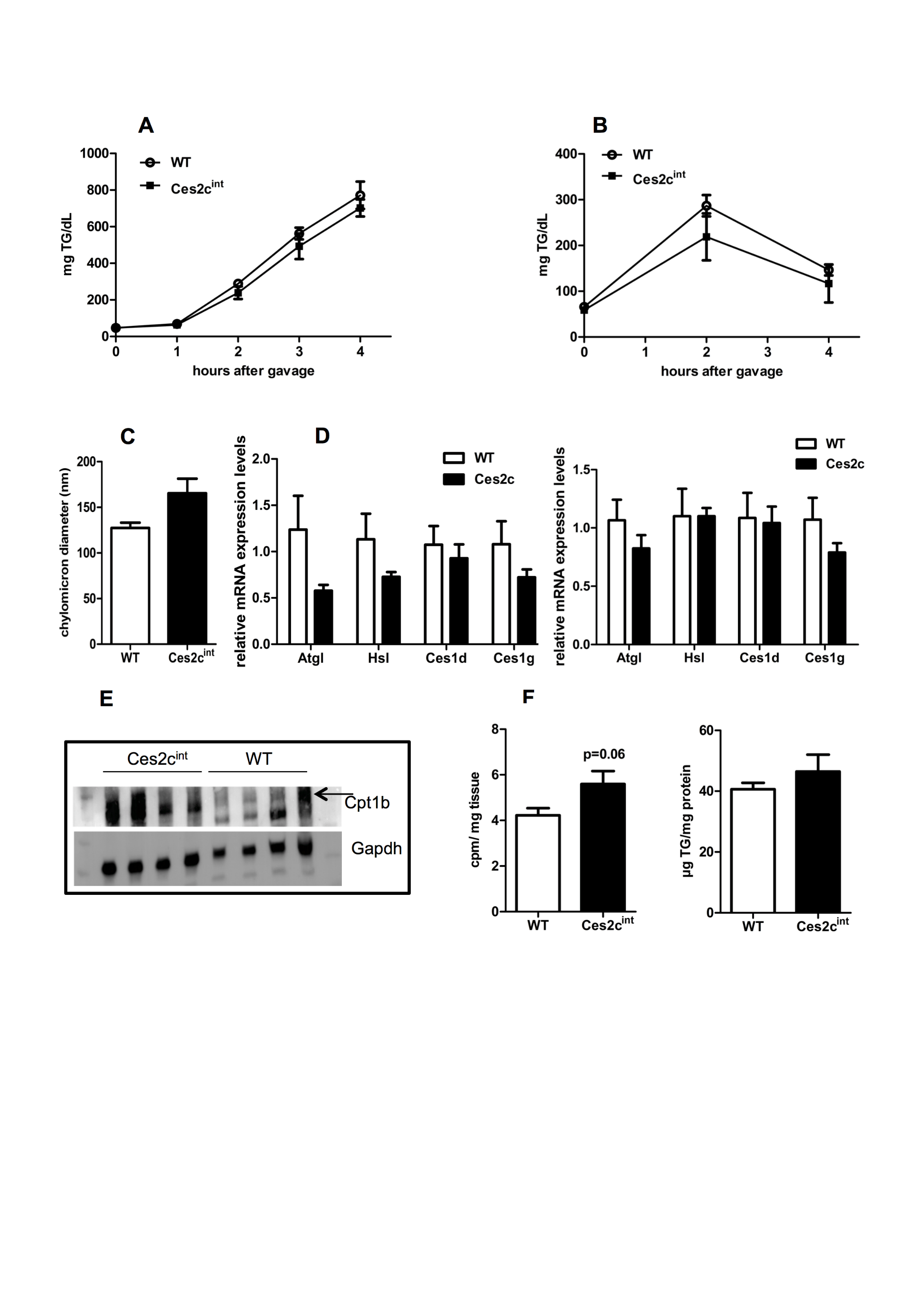
**

**Supporting FIG. 7. Chylomicron synthesis and clearance in Ces2c^int^ mice on HFD.** Six-week-old mice fed a HFD (45 kJ% fat; 22.1 kJ/g) for 24 weeks. (A) Female Ces2c^int^ and WT mice fasted overnight followed by intraperitoneal injection of a LPL clearance inhibitor (tyloxapol), and gavaged with olive oil. Plasma was collected before and 1, 2, 3, and 4 hours post gavage to measure TG concentrations. (B) To examine postprandial TG clearance, Ces2c^int^ and WT mice were fasted overnight and challenged with an olive oil bolus. Plasma samples were taken before and 2 and 4 hours after the gavage, and TG levels were determined (n = 6). (C) apoB_48_ lipoprotein particle size was measured by light scattering (n = 5). (D) mRNA expression levels of lipases were examined in the proximal (left panel) and distal small intestine (right panel) of HFD-fed Ces2^int^ mice and compared to controls (n = 5). (E) Western blot analysis of Cpt1b expression in *M. quadriceps* from mice on HFD for 24 weeks (n = 4). (F) OFTTs with [^3^H]-TG as tracer (left panel) or without a tracer (right panel) were performed with 14-week-old chow-fed mice. After 3 hours, *M. quadriceps* were excised, radioactivity levels (left panel) or TG content (right panel) were measured (n = 5) (n = 3). Data represent mean + SEM. Statistical significance was determined by student 2-tailed *t* test (**P* < 0.05; ***P* < 0.01; ****P* < 0.001). Abbreviation: HFD, high fat diet; LPL, lipoprotein lipase; TG, triglyceride.

**Supporting Experimental Procedures**

**Cloning and expression in COS-7 cells:** Ces2c carries a N-terminal signal sequence, that mediates localization to the ER and a C-terminal four amino acid long ER retention signal sequence (HREL). To ensure the localization of Ces2c to the ER, a C-terminally FLAG-tagged Ces2c mutant was generated. The ER retention signal was C-terminally attached to the FLAG-tag, to prevent mislocalization. Ces2c (NM_145603.2; Q91WG0) was amplified in two consecutive steps from liver cDNA. The following primers were used to amplify Ces2c CDS lacking the ER retention signal: Ces2c1, 5′TTA CGT GCG GCC GCC CAC CAT GAC ACG GAA CCA ACT ACA TAA C -3′ (forward) and 5′CCG GTT TCT AGA CTT GTC CTG AGA AGC CTT TAG C-3′ (reverse). Next, primers containing the FLAG-tag sequence followed by the ER retention sequence and a stop codon were used to amplify Ces2c: Ces2c2, 5′TTA CGT GCG GCC GCC CAC CAT GAC ACG GAA CCA ACT ACA TAA C-3′ (forward), 5′-CCG GTT GAT ATC CTA AAG CTC CCT GTG CTT GTC ATC GTC GTC CTT GTA A-3′ (reverse). PCR products were ligated to compatible restriction sites of the expression vector pFLAG-CMV-5.1 (Merck, Darmstadt, Germany). COS-7 cells (SV-40 transformed monkey embryonic kidney cells; ATCC, catalog no. CRL-1651) were transfected with recombinant DNA complexed to metafectene (Biontex Laboratories GmbH, Muenchen, Germany) in FCS-free medium. 4 hours after the transfection, the medium was changed to serum-containing medium. Cells were harvested 24 hours post transfection, and cell lysates were generated by brief sonication in buffer A (0.25 M sucrose, 1 mM EDTA, 1 mM dithiothreitol, 20 µg/mL leupeptin, 2 µg/mL antipain,1 µg/mL pepstatin; pH 7.0). CES2 plasmid (a kind gift from Richard Lehner) and LacZ/HisMax were expressed in Expi293 cells using the commercially available ExpiFectamin 293 transfection Kit (ThermoFisher Scientific, Waltham, MA). The transfection was carried out according to the manufacturer manual. Cells were harvested 48 hours post-transfection and cell lysates were generated by brief sonication in buffer A.

To stably overexpress FLAG-tagged Ces2c in COS-7 cells, a lentivirus was generated using the Lenti-X system (Takara Bio Europe, Saint-Germain-en-Laye, France). Full-length Ces2c CDS harboring a C-terminal FLAG-tag before the ER-retention signal was cloned into the pLVX-IRES-Puro vector (Takara Bio Europe, Saint-Germain-en-Laye, France), transfected into HEK 293T cells and assembled into infectious virions. These infectious virions were used to stably transduce COS-7 cells.

**DGH/TGH activity assay in cell and tissue lysates, stereoselectivity and pH optimum:** Briefly, the DG substrate consisted of 1 mM rac-dioleoyl-glycerol, and the TG substrate consisted of 0.32 mM nonradioactive triolein and 10 μCi Trioleat [9,10-^3^H]/ml (PerkinElmer Vertriebs GmbH, Traiskirchen, Austria). The dried substrate was dissolved in assay buffer (100 mM KPi, 50 mM CHAPS) and sonicated 3 times for 30 seconds on ice. For each reaction, either 50 μg protein (cell lysates 1,000 *g* supernatant) or 30 μg protein (intestinal tissue lysates 10,000 *g* supernatant) in buffer A were incubated for 1 hour at 37°C under constant shaking with the micellar TG substrate. The reaction was stopped by the addition of 3.25 mL of methanol/chloroform/n-heptane (10/9/7, vol/vol/vol) and 1 mL 0.1 M potassium carbonate [pH 10.5]. To separate the phases, the samples were vortexed vigorously and centrifuged at 1,000 *g* for 10 minutes. 200 μl of the upper aqueous phase was used for liquid scintillation counting.

To determine whether Ces2c exhibits positional selectivity for TG hydrolysis, we performed TGH assays as described above and separated the products by TLC. The assay was terminated by extracting the lipids according to Folch’s method.^(2)^ Subsequently, lipids were separated by TLC using chloroform/acetone/acetic acid (92/8/1, vol/vol/vol) as solvent. Bands corresponding to FAs and the different MG and DG species were cut out, and the radioactivity was determined by liquid scintillation counting.

*In vitro* TGH activity assays were performed as described above with some minor modifications to determine the pH optimum of Ces2c TGH activity. For each reaction, 50 μg protein (cell lysates 1,000 *g* supernatant) in buffer A was mixed with different pH buffers (200 mM potassium acetate [pH 4-6]; KPi [pH 6-8]; Tris/HCL [pH 8-10]). The assays were performed as described above.

**pNPA/pNPV assays:** To measure esterase activity, spectrophotometric assays, using p-nitro phenyl esters of various chain length FAs as substrate, were performed. Short-chain esters are water soluble, and therefore their hydrolysis provides a measure of esterase activity. The assays are based on the release of p-nitrophenolat, which is measured spectrophotometrically at 405 nm.

Briefly, 3 mM pNPA or pNPV was dissolved in 100 mM KPi buffer. For each reaction, 100 μg protein (cell lysates 1,000 *g* supernatant) in buffer A was incubated for up to 5 minutes with the pNPA or pNPV substrate. The extinction was measured at either 405 nm (pNPA) or 405/620 nm (pNPV).

**Acylcarnitine hydrolase activity:** To measure acylcarnitine hydrolase activity, FA release from palmitoyl-carnitine was measured enzymatically. Briefly, 50 μg protein (cell lysates 1.000 g supernatant) in buffer A was incubated with the substrate for 30 minutes at 37°C. 2 mM palmitoyl-carnitine-chloride (Merck, Darmstadt, Germany) was used as substrate and prepared by sonication in assay buffer consisting of 200 mM KPi buffer [pH 7.0] containing 1 mM EDTA, 6.4 mM CHAPS, and 600 mM NaCl. The reaction was stopped by heat inactivation at 75°C for 10 minutes. The released FFAs were measured enzymatically from aliquots using the commercially available NEFA C Kit (Wako Chemicals, Neuss, Germany).

**FAO in cell culture:** Stably transduced COS-7 cells were seeded into 25-cm flasks (Greiner Bio-One, Kremsmuenster, Austria) to achieve confluence. To measure FAO, cells were serum starved overnight in DMEM 1 g/L glucose (ThermoFisher Scientific, Waltham, MA) containing 1% FCS (ThermoFisher Scientific, Waltham, MA) and 0.5 mM carnitine (Merck, Darmstadt, Germany). Following the overnight starve, the cells were additionally starved for 2 hours in the absence of glucose, glutamine, and serum in DMEM A14430 (ThermoFisher Scientific, Waltham, MA) containing 0.5 mM carnitine. To measure glucose oxidation, cells were starved for 2 hours in DMEM A14430. Incubation was initiated by addition of DMEM A14430 containing either 0.5 mM carnitnine, 100 µM PA and 0.4 µCi PA (^1-14^C) (Hartmann Analytic GmbH, Braunschweig, Germany) or 1 mM glucose and 0.3 µCi glucose (^14^C) (Hartmann Analytic GmbH, Braunschweig, Germany) per flask. Flasks were sealed with a rubber stopper (Kimble Chase, Vineland, NJ) fitted with a suspended center well (Kimble Chase, Vineland, NJ) containing whatman filter paper, saturated with NaOH, and incubated at 37°C. Reactions were terminated after 90 minutes with the injection of 0.1 mL 70% perchloric acid through the rubber stopper. CO_2_ was trapped for 2 hours at 37°C. Filter papers from the center wells were placed in scintillation vials and counted. Stock solutions containing radiolabeled PA or glucose were also counted to determine the specific activity and to calculate the extent of oxidation. To measure ASMs, the incubation medium was collected, centrifuged at 13.000 *g* for 10 minutes, and 200 µl of the supernatant were used for liquid scintillation counting. To normalize the protein content, cells were lysed in 0.3 N NaOH, 0.1 M SDS, and protein content was determined by BCA measurement (VWR International, Radnor, PA).

**FA uptake and FA incorporation studies:** FA uptake was measured in stably transduced COS-7 cells. Briefly, cells were incubated for 1 minute with 1 µCi OA. Afterward, they were washed 3 times with PBS and lysed in 0.3 N NaOH, 0.1 M SDS. An aliquot was used for liquid scintillation counting. For FA incorporation studies, COS-7 cells stably overexpressing recombinant FLAG-tagged Ces2c were seeded into 12 well plates and loaded with 400 μM OA and 1 µCi OA [^9,10-3^H] (Hartmann Analytic GmbH, Braunschweig, Germany) for 20 hours to induce LD formation. After the labeling period, the cells were starved for 4 hours and 6 hours in DMEM A14430 containing 2% FFA-free BSA. Total lipids were extracted by hexane/isopropanol (3:2) after the labeling and designated starve periods, and TLC was carried out using hexane/diethyl ether/acetic acid (70:29:1) as solvent. TG bands were excised, and the amount of radioactivity was measured by liquid scintillation counting. To normalize the protein content, cells were lysed in 0.3 N NaOH, 0.1 M SDS, and protein content was determined by BCA measurement.

**Generation transgenic animals and genotyping:** In brief, full-length Ces2c cDNA (amplified from liver cDNA) with a FLAG-tag fused at the C-terminus followed by the ER-retention signal HREL was cloned into the *AgI/Xho*I sites of the 12.4-kb Villin-delta ATG plasmid. The transgene DNA was excised with *Pme*I, purified, and injected into the pronuclei of fertilized eggs of C57BL/6N mice. The transgene was detected in tail genomic DNA by conventional PCR (5′ GGG AGG GGT ATG TTT TAA GTG CTG GG 3′ forward, 5′TCG CAG TGG TCC TAC AGG AGG T 3′ reverse, PCR product 520 nt). Transgenic founders, which originated from a C57BL/6N background, were backcrossed four to five times on the C57BL/6J background to perpetuate the transgenic lines.

**HFD feeding study and food intake:** For HFD feeding, all mice were fed standard mouse chow *ad libitum* from weaning to 5 weeks of age; at that time, the diet was switched to a HFD for 16 weeks. Once weekly, the food was refreshed, and body weight measurements were taken. To monitor food intake and fat absorption, the mice were single housed. Individual food intake was measured by pellet weight every second day for 2 weeks. Body mass composition of each animal was determined with a calibrated miniSpec NMR analyzer (Bruker Optics, Billerica, MA).

**Fat absorption, feces output and analysis:** Fat absorption was determined by the sucrose polybehenate method as described.^(3)^ Briefly, single-housed mice were fed the test diet, which contained 5% sucrose polybehenate ester and 16% fat from safflower oil, for 2 days, after which their cage bedding was changed and fecal samples were collected on 2 subsequent days. The FA content and composition of the fecal pellet were determined by gas chromatography (GC-MS) by the University of Cincinnati Mouse Metabolic Phenotyping Core (MMPC). Fecal output was measured on 5 consecutive days. Mice were single housed, their cage bedding was changed every morning, and feces was collected, dried, and weighed. For fecal energy content measurement, feces of single-housed mice were collected every day for 2 weeks. The feces were dried and grounded. For the measurement, 1 g of feces was pressed into a tablet and burned in an adiabatic oxygen bomb calorimeter C4000 A (IKA Analysentechnik, Stauffen, Germany). Each measurement was performed in triplicates.

**Dietary fat uptake in the small intestine and clearance in muscle:** Fat absorption along the length of the small intestine was assessed as previously described. Mice were fasted overnight (12 hours) and received an oral gavage of 1 µCi ^3^H Triolein in 100 µl olive oil. After 3 hours, *M. quadriceps* and the small intestine were excised. The small intestine was cut between the base of the stomach and the cecal junction, flushed with 0.5 mM sodium taurocholate in PBS, and cut into 2-cm segments. *M. quadriceps* was lysed in 2.5 ml of 0.3 N NaOH, 0.1 M SDS, while each intestinal segment was lysed with 0.5 mL 0.3 N NaOH, 0.1 M SDS at 65°C, transferred to scintillation vials containing 5 mL scintillation liquid, and measured.

**Metabolic phenotyping:** For metabolic phenotyping, mice were housed in a laboratory animal monitoring system (Phenomaster, TSE Systems, Bad Homburg von der Höhe, Germany), enabling the continuous measurement of locomotor activity, oxygen consumption, and carbon dioxide elimination of the body. Prior to metabolic cage housing, the mice were familiarized to single housing for a week and drinking flasks for at least 24 hours. Mice were acclimatized for 1 day before monitoring the parameters and allowed *ad libitum* access to food and water. Data were separated by the light (14-hour)/dark (10-hour) cycle and averaged over 6 cycles for each mouse. Results are shown as means over animal groups. TEE, RMR, and AEE were calculated as described.^(1)^ Body temperature was measured in conscious mice using a rectal probe RET-3 (Physitemp, Clifton, NJ).

**Liver histology:** For conventional light microscopy, livers from HFD-fed mice were fixed in 4% neutral buffered formaldehyde solution for 24 hours. Cryosections were prepared and stained with Oil Red O according to standard protocol.

**Tissue lipid measurement:** Tissue lipids were extracted according to the method of Folch et al.^(2)^ The extracted lipids were dried in a stream of nitrogen, resuspended in 2% Triton X-100, and redissolved by brief sonication. TG levels were measured using the Infinity Triglycerides Reagent (ThermoFisher Scientific, Waltham, MA). To determine tissue protein, the remaining protein pellets were dried and afterward lysed in lysis buffer (0.3 N NaOH, 0.1 M SDS). Subsequently the protein content was measured using the BCA reagent (ThermoFisher Scientific, Waltham, MA).

**Postprandial lipoprotein secretion and FPLC analysis:** To determine postprandial lipoprotein secretion, mice were kept either on chow-diet or HFD. After 12 hours of fasting, blood was collected (time 0), an intragastric bolus of lipids (200 µl of olive oil) was administered, and blood samples were collected at indicated time points from the orbital before and after the gavage. Plasma TG levels were determined as described in Plasma Chemistry. Lipoprotein fractions from plasma of a pool of 200 µl per group (collected 3 hours post gavage) were separated by fast protein liquid chromatography equipped with a Superose 6 column (Amersham Biosciences, Piscataway, NJ). TG and TC concentrations in the isolated fractions were determined spectrophotometrically (DiaSys, Holzheim, Germany).

**Chylomicron secretion:** For analysis of chylomicron secretion, mice were maintained on chow-diet for 14 weeks. After a 12-hour fast, blood was collected (time 0). Mice were then weighed and received an intraperitoneal injection with 500 mg/kg tyloxapol (Triton WR-1339, Merck, Darmstadt, Germany) to block lipoprotein lipase activity. Thirty minutes after the injection, mice were gavaged with 200 µl of olive oil, and blood samples were collected from the orbital plexus at 1, 2, 3, and 4 hours after the gavage. Plasma TG levels were determined as described in Plasma Chemistry. For chylomicron size measurement, chylomicrons were isolated by density gradient centrifugation as described with some modifications.^(4)^ Briefly, 0.4 mL of plasma was mixed with 2.5 mL PBS containing potassium bromide (density 1.2 g/mL) and transferred to SW41 tubes. The mixture was overlayed with 0.9% NaCl and centrifuged for 45 minutes in an SW41 rotor (Beckman, Fullerton, Canada) (40,000 *g*, 4°C). Chylomicrons (visible as white layer at the top of the tube) were collected and analyzed by dynamic light scattering using a Zetasizer Nano ZS (Malvern Instruments Ltd., Malvern, UK) at 25°C according to manufacturer’s instructions. Measurements were performed as triplicates, averaged, and indicated as mean hydrodynamic diameter in nanometer scale (Z-Average D_nm_).

Chylomicron clearance: To examine chylomicron clearance into liver and muscle, chow-fed mice were fasted for 12 hours. Afterwards, an intragastric bolus of lipids (200 µl of olive oil) was administered. After 3 hours, livers and *M. quadriceps* were excised and TG content was determined as described in tissue lipid measurements.

Protein method: Livers and *M. quadriceps* of *ad libitum*–fed mice were homogenized in buffer A (0.25 M sucrose, 1 mM EDTA, 1 mM dithiothreitol, 20 µg/mL leupeptin, 2 µg/mL antipain,1 µg/mL pepstatin; [pH 7.0]) on ice using an Ultra Turrax (IKA Analysentechnik, Stauffen, Germany). Intestinal protein was isolated from different parts of the small intestine using glass beads (Carl Roth GmbH, Karlsruhe, Germany) or MagNA Lyser green beads (Roche Diagnostics, Rotkreuz, Germany) according to manufacturer’s protocol, containing extraction buffer (25 mM HEPES/KOH [pH 7.4], 0.25 M sucrose, 0.5 mM EDTA) or buffer A with protease and phosphatase inhibitors added. The homogenates were centrifuged at 1.000 *g* at 4°C for 10 minutes, and the infranatants were collected.

For western blotting, 80 µg of liver protein, 30 µg of *M. quadriceps* or 20 µg intestinal protein was separated by either 7.5%, 10%; 5-12% or 3-12% SDS-PAGE followed by western blot analysis using PVDF membranes (Carl Roth GmbH, Karlsruhe, Germany) and CAPS buffer (10 mM 3-cyclohexylamino-1-pro- panesulfonic acid, 10% methanol) for protein transfer. Blots were probed using either specific antibodies against Akt (C67E7, Cell Signaling Danvers, MA), ApoB (ab20737, Abcam, Cambridge, UK), mouse Carboxylesterase 2/CES2 (AF5280, R&D Systems, Minneapolis, MN), CPT-1 (sc-98834, Santa Cruz, Santa Cruz, CA), FLAG-HRP (A8592, Merck, Darmstadt, Germany), GAPDH (2118S, Cell Signaling, Danvers, MA), His (18184, Abcam, Cambridge, UK), mTOR (2972, Cell Signaling Technology, Inc., Danvers, MA), p-Akt/PKB (Ser473) (9271S, Cell Signaling Technology, Inc., Danvers, MA), P70S6K (2708S, Cell Signaling Technology, Inc., Danvers, MA), p-P70S6K (Thr389) (9205, Cell Signaling Technology, Inc., Danvers, MA) or phospho-mTOR (Ser2481) (2974S, Cell Signaling Technology, Inc., Danvers, MA). Specifically bound immunoglobulins were detected in a second reaction using horseradish peroxidase-conjugated anti-rabbit or anti-mouse IgG antibody and visualized with BioRad Gel Doc/Chemi Doc Imaging System and analyzed using ImageLab Software.

**RNA extraction and quantitative RT-PCR:** Tissue RNAs were extracted using Trizol reagent (ThermoFisher Scientific, Waltham, M). For cDNA synthesis, 1 µg of total RNA was reverse transcribed using random primers (ThermoFisher Scientific, Waltham, MA) and superscript II reverse transcriptase (Invitrogen, Carlsbad, CA). RT-PCR was conducted using SYBR Green (ThermoFisher Scientific, Waltham, MA) and the ABI-StepOnePlus detection system (ThermoFisher Scientific, Waltham, MA). Relative mRNA levels were quantified by DDCt method with 36B4 as reference gene.

**Targeted lipidomic analysis:** Weighed intestinal tissue explants (20-60 mg) were transferred to 2-mL Safe-Lock PP tubes and extracted according to Matyash et al*.*^(5)^ In brief, samples were homogenized using two 6-mm steel beads on a Mixer Mill (Retsch, Haan, GER; 2 × 15 seconds, frequency 30/second) in 700 µl MTBE/MeOH (3/1, vol/vol) containing 500 pmol butylated hydroxytoluene, 1% acetic acid, and 100 pmol of internal standards , 17:0 MG, 14:0-14:0 DG; Avanti Polar Lipids, Alabaster, AL) per sample. Total lipid extraction was performed under constant shaking for 30 minutes at RT. After addition of 140 µl dH2O and further incubation for 30 minutes on RT, samples were centrifuged at 1,000*g* for 15 minutes to establish phase separation. 500 µl of the upper, organic phase was collected and dried under a stream of nitrogen. Lipids were resolved in 500 µl 2-propanol/methanol/water (7/2.5/1, vol/vol/vol) for UPLC-MS analysis. The residual extraction mixture was dried for protein determination and lysed using 500 µl NaOH/SDS (0.3 N/0.1%). Chromatographic separation was modified after Knittelfelder et al.^(6)^ using an ACQUITY-UPLC system (Waters Corporation, Milford, MA), equipped with a Luna omega C18 column (2.1 x 50 mm, 1.6 µm; Phenomenex) starting a 20-minute linear gradient with 80% solvent A (MeOH/H_2_O, 1/1, vol/vol; 10 mM ammonium acetate, 0,1% formic acid, 8 µM phosphoric acid). The column compartment was kept on 50°C. An EVOQ Elite triple quadrupole mass spectrometer (Bruker, Billerica, MA) equipped with an ESI source was used for detection. Lipid species were analyzed by selected reaction monitoring ([MNH4]+ to [RCOO+58]+ of the respective esterified FA; DG: 15 eV, 50 ms; MG: 11 eV, 60 ms; the resolution of Q1/Q3 was set to 0.7). Data acquisition was done by MS Workstation (Bruker, Billerica, MA). Data were normalized for recovery, extraction, and ionization efficacy by calculating analyte/ISTD ratios (AU) and expressed as AU/g tissue or AU/mg tissue protein.

**Analyses of OCRs:** Fresh small intestine was collected and rinsed with PBS. The small intestine was cut into three equal pieces (proximal intestine, middle intestine, distal intestine), and 2-cm pieces were briefly homogenized in 500 µl ice-cold 250 mM sucrose using Ultra-Turrax Homogenizer and passed through a 100-mm cell strainer. High-resolution respirometry, employing a polarographic oxygen sensor in a two-chamber oxygraph (OROBOROS Instruments, Innsbruck, Austria), was used to measure OCRs. 25 µl of intestinal homogenate was incubated in respiration buffer (125 mM sucrose, 20 mM K-Tes [pH: 7.2], 2 mM MgCl2, 1 mM EDTA, 4 mM KH2PO4, 3 mM malate, and 0.1% essentially FA-free BSA) and measured at 37°C. Respiration was analyzed in the presence of 1 mM Adenosine diphosphate (ADP) and 0.2 µM cytochromC by adding substrates successively upon reaching a steady state (50 µM Octanoyl-CoA L-Carnithin (C-8 carnitine), 2.5 µM Oligomycine (Oligo). Data were analyzed using DatLab software (version 5.1.1.91). OCRs were calculated per milligram of protein.

1) van Klinken JB, van den Berg SAA, Havekes LM, Willems Van Dijk K. Estimation of activity related energy expenditure and resting metabolic rate in freely moving mice from indirect calorimetry data. PLoS One 2012;7:e.

2) Folch J, Lees M, Stanley GHS. A simple method for the isolation and purification of total lipides from animal tissues. J Cell Biol 1957;226:497-509.

3) Jandacek RJ, Heubi JE, Tso P. A novel, noninvasive method for the measurement of intestinal fat absorption. Gastroenterology 2004;127:139-144.

4) Sachdev V, et al*.* Novel role of a triglyceride-synthesizing enzyme: DGAT1 at the crossroad between triglyceride and cholesterol metabolism. Biochim Biophys Acta 2016;1861:1132-1141.

5) Matyash V, Liebisch G, Kurzchalia TV, Shevchenko A, Schwudke D. Lipid extraction by methyl- *tert* -butyl ether for high-throughput lipidomics. J Lipid Res 2008;49:1137-1146.

6) Knittelfelder OL, Weberhofer BP, Eichmann TO, Kohlwein SD, Rechberger GN. A versatile ultra-high performance LC-MS method for lipid profiling. J Chromatogr B Analyt Technol Biomed Life Sci 2014;951–952:119-128.
